# Implementation and fidelity of reactive surveillance and response strategies for malaria elimination: a systematic review and meta-analysis

**DOI:** 10.1136/bmjph-2024-001180

**Published:** 2025-11-13

**Authors:** Win Han Oo, Nilar Aye Tun, Kaung Myat Thu, Julia C Cutts, Win Htike, Ei Phyu Htwe, May Chan Oo, Aung Khine Zaw, Paul Anthony Agius, Freya J I Fowkes

**Affiliations:** 1 Disease Elimination, Burnet Institute, Melbourne, Victoria, Australia; 2 Health Security and Pandemic Preparedness, Burnet Institute Myanmar, Yangon, Yangon, Myanmar; 3 Melbourne School of Population and Global Health, The University of Melbourne, Melbourne, Victoria, Australia; 4 Faculty of Health, Deakin University, Melbourne, Victoria, Australia; 5 Department of Epidemiology and Preventive Medicine, Monash University, Melbourne, Victoria, Australia

**Keywords:** Sentinel Surveillance, Disease Notification, Disease Hotspot, Disease Eradication, Public Health

## Abstract

**Introduction:**

To achieve malaria elimination, case-based surveillance and response systems have been developed in endemic countries. This study systematically reviews the different types of reactive surveillance and response strategies for malaria, how they are being implemented, and their implementation fidelity, across different malaria elimination settings to inform reactive surveillance and response guidelines.

**Methods:**

A systematic review of published and grey quantitative and qualitative studies investigating different reactive surveillance and response strategies was conducted. Five databases (PubMed, Web of Science, Scopus, African Index Medicus and Latin American and Caribbean Health Sciences Literature) were searched in all years up to 16 June 2025 with no restrictions on language or publication date. Meta-analyses were performed to obtain pooled estimates of each implementation fidelity outcome of reactive surveillance and responses. Sub-group analyses of geographical regions were performed.

**Results:**

69 studies (33 in the Asia-Pacific, including 16 in Greater Mekong Subregion, 34 in Africa and two in South America regions) were included in the review; of which 43 were included in the meta-analysis. The ‘1-3-7’ strategy is a common reactive surveillance and response strategy developed in China and adopted by Greater Mekong Subregion countries. In Africa, many countries undertook reactive case detection alone as their reactive surveillance and response strategy (19/34 studies). In general, the implementation fidelity measured by completeness and timeliness of each step of reactive surveillance and responses was 100% in China (completeness and timeliness of case investigation: 100% (95% CI 100% to 100%) and 97% (95% CI 93% to 100%), respectively). Lowest fidelities of reactive surveillance and responses were observed in studies conducted in Africa and Greater Mekong Subregion (timeliness of case investigation: 56% (95% CI 26% to 83%) in Africa and 57% (95%CI 37% to 77%) in Greater Mekong Subregion).

**Conclusion:**

The implementation fidelity of reactive surveillance and responses varies within and across countries and regions. This may be due to differences in regional, national and sub-national health system capacity and infrastructure. Each endemic country implementing the malaria elimination programme should reevaluate, refine, improve and strengthen the ongoing strategy which is fit-for-purpose of the national health system capacity considering the global and regional evidence.

**Trial registration number:**

International prospective register of systematic reviews (PROSPERO), CRD42021249857.

**WHAT IS ALREADY KNOWN ON THIS TOPIC**

- Many malaria-endemic countries recently apply reactive surveillance and response strategies to strengthen case-based surveillance system in the malaria elimination programme.

**WHAT THIS STUDY ADDS**

- In order to improve malaria reactive surveillance and response strategies, a systematic review and meta-analysis was performed with the synthesis of regional evidence. The implementation and fidelity of malaria reactive surveillance and response strategies vary according to geographical region. Its fidelity was optimal in China where the 1-3-7 reactive surveillance and response strategy was developed, but suboptimal in Greater Mekong Subregion countries, and in studies conducted in many African countries where reactive case detection alone was implemented.

**HOW THIS STUDY MIGHT AFFECT RESEARCH, PRACTICE OR POLICY**

- Endemic countries aiming for malaria elimination may tailor 1-3-7 reactive surveillance and response strategy fit-for-purpose of the national health system capacity considering the global and regional evidence derived from this paper.

## Introduction

 Many countries have set malaria elimination goals: Asia-Pacific countries^1^ and eight African countries aim to eliminate malaria by 2030.^2^ Once the number of malaria cases has reduced to a particular low level, typically annual parasite incidence of less than one per 1000 at-risk population, in a particular area or country in the control phase, the priorities and activities of a malaria programme need to be reorientated in order to achieve elimination.^3^ In the malaria elimination phase, surveillance should be enhanced to ensure that every malaria case is detected and reported in a timely manner. Targeted measures for both parasites and vectors should be implemented effectively in order to interrupt local transmission.^3^ Therefore, it is essential to establish case-based surveillance and response systems to ensure that every malaria case is investigated, to understand risk factors and to eliminate foci of transmission.^4^ In this system, surveillance must become increasingly granular, allowing the identification, tracking and classification of all malaria cases and implementation of appropriate response activities.^3 4^

The Global Technical Strategy for Malaria (2016–2030) endorsed at the 2015 World Health Assembly laid out the strategic framework for elimination with three pillars, two of which emphasise surveillance, where Pillar two explicitly mentioned components of malaria surveillance and response programmes.^5^ The WHO recommends that national malaria programmes define a suitable schedule in case-based surveillance and response systems for case detection and notification, case investigation and classification, focus investigation and responding, and conduct regular monitoring and evaluation of their surveillance systems.^4^ These activities are interconnected and are collectively referred to as ‘case investigation, foci investigation and response’ or herein as ‘ReActive Surveillance and Response (RASR) strategies’.

The RASR strategy is only comprehensive if all three steps of (1) case notification, (2) case investigation and classification and (3) focus investigation and response are completed. Globally, different RASR strategies have been developed and implemented in malaria-endemic countries to achieve malaria elimination.^6^ Another malaria surveillance strategy of test-treat-track is distinct from the RASR strategy, in which ‘test’ entails ‘Every suspected malaria case should be tested’, ‘treat’ means ‘Every confirmed case should be treated with quality-assured antimalarial medicine’ and ‘track’ refers to ‘Every malaria case should be tracked in a surveillance system’.^7^

A common RASR strategy in the Asia-Pacific is the 1-3-7 strategy, which entails case notification within 1 day, case investigation within 3 days, and focus investigation and appropriate public health responses within 7 days.^8^ This 1-3-7 strategy, developed in 2010 and rolled out nationwide in China in 2012,^9^ contributed to the successful elimination of malaria in China in 2021. While China’s health infrastructure has enabled successful implementation of the 1-3-7 strategy, it is unclear how this strategy may be translated or adapted to different malaria transmission and elimination settings. Another common activity included in the focus investigation and response step is reactive case detection (RACD).^6 10^ After an index case has been investigated and classified, RACD is implemented^11^—which is an active case detection strategy that tests and treats malaria infection of people geographically proximal (hot spots) to the index case (confirmed symptomatic cases that are identified through passive surveillance) or among populations who share the same characteristic (hot pops) such as co-travellers and co-forest goers.^12^ RACD is implemented as per the assumption that malaria cases (either asymptomatic or symptomatic) could cluster around the index case geographically or population characteristically. RACD’s end goal is to detect any malaria cases, symptomatic or asymptomatic, linked to the index case and respond in a timely manner to prevent onward malaria transmission. Therefore, RACD may be perceived as more effective and cost-effective than applying mass screening and treatment or reactive drug administration approaches. A recent systematic review^13^ compared the effect of two reactive strategies, ‘reactive case detection and treatment’ and ‘reactive drug administration’, on malaria transmission reduction, finding the latter might be more effective but with limited certainty of evidence. Nevertheless, there is no consensus on the effectiveness of each of these RACD strategies. Further, their cost-effectiveness is yet to be evaluated globally, and there are no international standard guidelines and procedures on how to perform RACD as a part of an RASR strategy.

Here, we systematically review the different RASR strategies, how they are being implemented and their implementation fidelity across different malaria elimination settings in order to inform RASR guidelines. Specifically, meta-analyses of the completeness and timeliness in each step of RASR by region were undertaken. Strategies implemented in high-performing regions could be learnt and adopted in other regions for better malaria surveillance and responses which may contribute to achieving malaria elimination globally.

## Methods

A systematic review of published and grey quantitative and qualitative studies investigating different RASR strategies was conducted in Covidence. The protocol for this review was prospectively registered with International Prospective Register of Systematic Reviews (protocol number CRD42021249857) and is summarised in online supplemental material S1. It adheres to the Preferred Reporting Items for Systematic Reviews and Meta-Analyses (2020 Checklist)^14^ and the Meta-analysis of Observational Studies in Epidemiology guidelines for systematic reviews.^15^

### Search strategy and selection criteria

All interventional and observational studies conducted in malaria-endemic areas (control and elimination as defined by the malaria atlas project^16^) containing data describing the outcomes of interest were eligible for inclusion. PubMed, Web of Science, Scopus, African Index Medicus and Latin American and Caribbean Health Sciences Literature initially were searched, all years up to 30 July 2021, with an updated search of databases between 30 July 2021 and 16 June 2025, with no restrictions on language (online supplemental material S6). Boolean search methods were applied with the following terms (malaria OR plasmodium OR vivax OR falciparum) AND (‘reactive surveillance’ OR ‘case detection’ OR ‘foci investigation’ OR ‘RACD’ OR ‘1-3-7’). The searches included peer-reviewed publications and grey literature such as evaluation reports, policy guidelines and strategy documents. The retrieved literature was first screened by title and abstract, then screened a second time by reading the full text (online supplemental material S2). Screening was done by two authors independently with discrepancies resolved through discussion with a third author.

### Data extraction

Data from the included papers were extracted using the data extraction proforma (online supplemental material S3) independently by three authors. If further information that was not featured in the literature was required, authors were contacted up to two times via email.

### Outcomes

The outcomes of interest were elements of the implementation of each step of RASR such as (1a) case notification, (1b) case investigation and classification and (1c) focus investigation and responses including RACD. The outcomes of interest also included implementation fidelity of malaria RASR measured by levels of completeness and timeliness of (2a) case notification, (2b) case investigation and (2c) RACD as per WHO guidelines,^11^ calculated by formulae (online supplemental material S4) and stratified by geographical region (Greater Mekong Subregion (GMS), China, other Asia-Pacific, Africa and South America).

### Risk of bias

Risk of bias in non-randomised quantitative studies was assessed using The Risk Of Bias In Non-randomised Studies—of Interventions assessment tool.^17^ For randomised studies, the Cochrane Collaboration’s tool for assessing risk of bias was used.^18^ Qualitative studies were assessed using the Critical Appraisal Skills Programme checklist for qualitative studies.^19^

### Synthesis and meta-analysis

Meta-analyses were performed through the application of DerSimonian–Laird random-effects estimation to obtain overall and geographical region-specific pooled estimates for each completeness and timeliness outcome^20^ and presented in forest plots. Given that in several studies, outcome proportions were close to the margin (ie, 0% or 100%) and that in subgroup analyses the number of studies was relatively small, the Freeman–Tukey double arcsine transformation provided more stable variance estimation. In forest plots, study-specific 95% CIs were estimated using the exact method. Contingent on a sufficient minimum number of studies, I-squared statistics (I^2^) were produced to quantify between-study and between-group heterogeneity,^21^ and both 95% CI and predictive intervals (PI) (ie, 95% CIsintervals incorporating the study-specific random effects/heterogeneity) for each pooled estimate were estimated in summary forest plots. PI represents the lower and upper bounds for prevalence which would be expected for any new study (ie, dispersion about true outcome prevalence of new studies). Applying Stata version 16.1 (StataCorp, College Station, TX, USA), user-written/wrapper programme *metaprop_one* was used to implement the random effect meta-analyses.

### Patient and public involvement

It is not applicable given this is the systematic review of published and grey literature.

### Ethics approval and consent to participate

Not applicable. There was no primary data collection from or intervention to any human subjects.

## Results

### Identification and description of included studies

**Figure 1:**
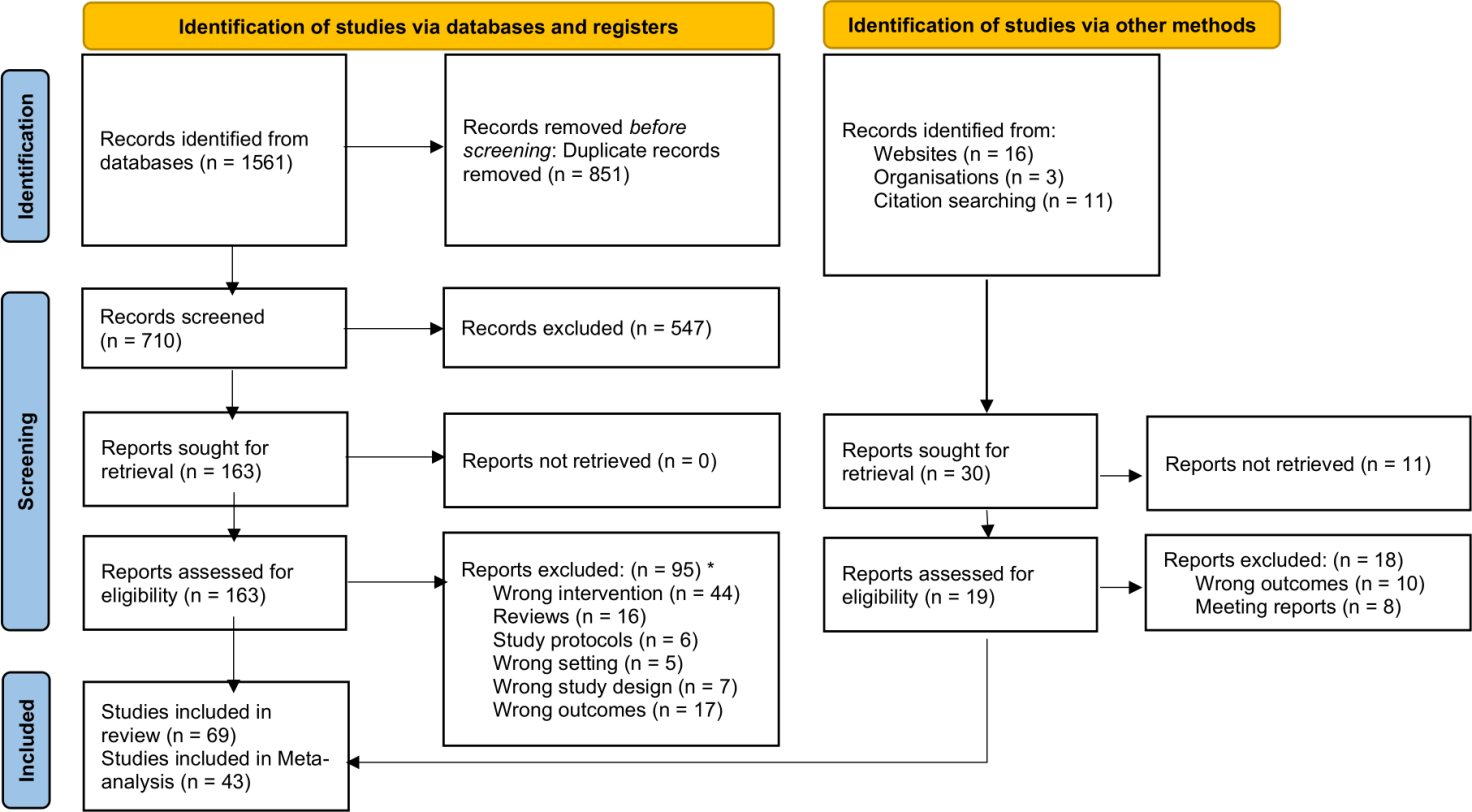
Preferred Reporting Items for Systematic Reviews and Meta-Analyses flow diagram. From Page MJ, McKenzie JE, Bossuyt PM, Boutron I, Hoffmann TC, Mulrow CD, *et al*. The PRISMA 2020 statement: an updated guideline for reporting systematic reviews. BMJ 2021;372:n71. doi: 10.1136/bmj.n71. For more information, visit: http://www.prisma-statement.org/. There were 1561 records across five databases (522 from PubMed, 641 from Web of Science, 352 from Scopus, 11 from African Index Medicus and 35 from Latin American and Caribbean Health Sciences Literature) based on the search strategy. Then, 851 duplicates were removed, leaving 710 records before any screening was done. After screening by title and abstract, 163 records (studies) were left as 547 records were excluded as per the reasons mentioned in the review protocol (online supplemental material S1). Then, a full 163 papers were retrieved to be assessed for eligibility by reading the whole manuscript. At that stage, 95 papers were excluded* leaving 68 records to be included in the review. In addition to the database search, citations of the 68 papers included in the review, and websites and organisation databases were searched for relevant records. 30 reports were sought for retrieval from which 19 reports were successfully retrieved and assessed for eligibility. From this stream, one report was finally included in the review, making a total number of 69 papers (68 from databases + one from other methods) for inclusion. *Excluded papers with reasons are available in online supplemental material S2.

In total, 1561 and 30 records published between 4 October 2010 and 17 January 2025 were identified from databases and grey literature sources, respectively. After screening, 69 papers were eligible for inclusion in the systematic review (figure 1). All included studies (online supplemental material S5) were reported in English. Briefly, the 69 included studies (detailed in online supplemental table S1) were conducted in hypo/mesoendemic settings approaching malaria elimination in 17 countries across the Asia-Pacific Region (33 studies including 16 studies in GMS countries), 11 countries in Africa Region (34 studies) and two countries in the South American Region (two studies). 43 studies reported data on one or more steps of the 1-3-7 strategy (27, 15 and 1 studies in Asia-Pacific, Africa and South American Regions, respectively), while 26 studies reported on the implementation of RACD (6, 19 and 1 studies in Asia-Pacific, Africa and South American Regions, respectively) (online supplemental table S1). The risk of bias of the included studies was assessed as moderate for randomised studies (5/5) and moderate-to-serious for the majority of non-randomised studies (55/64).

#### Implementation of reactive surveillance and response strategies

RASR strategies were implemented through case notification, case investigation and classification, and foci investigation and responses including RACD as a part of focus response (table 1).

**Table 1:** Implementation of reactive surveillance and response strategies

Step of RASR	Activities	Responsible person	Method/tool of execution	Time schedule
Case notification^922^,^49^	Notification of the detected malaria case to the responsible authorities in the health department	Frontline service provider such as CHW or health centre staff	Standalone tool or combination of Paper-based reporting^28 34 42 48^ Direct phone call^29 36 37 42 47^ Short message Service alert^3643^,^45^ Notification via web-based application^22 23 25 28 31 48^	Within 1 day is the standard.^922^,^46^ Vietnam,^47 48^ Thailand and Indonesia^30^ set 2, 3 and 30 days, respectively.
Case investigation and classification^922^,^25 27 29^	Case investigation and GPS coordinates collection Case classification as per WHO guidelines (indigenous, imported, introduced, induced, relapse and recrudescent)	Malaria programme focal staff at commune, township or district CHW (only two studies in Zambia^44 64^	Administering the questionnaire using a set form Recording GPS coordinates^42 45 48 52 63^	Within 3 days is the standard in 1-3-7 approach. Eswatini (formerly Swaziland)^29 55^ set 2 days and Vietnam^48^ set either 2 or 3 days depending on their 2-3-7 or 2–7 strategy, while Indonesia^30^ set 30 days.
Reactive Case Detection (RACD)^923 26 30^,^32 35^	RACD in ‘hotspots’ defined geographically or “hotpops” defined demographically	RACD team normally includes: Microscopist or lab technician Surveillance officer Midwife or basic health staff CHW or village leader Field coordinator	Malaria diagnostic tool being used as standalone or combination of Rapid diagnostic test (RDT)^35^,^3742^ Microscopy^26 41 42 48 49 52 65 77 79 80^ PCR^9 26 41 42 49 52 56 60 65 66 69 70 72 73 77 79 80 87 93^ Loop mediated isothermal amplification^55 68 69 79^	Within 7 days is the standard in 1-3-7 approach Ethiopia^73^ set 2 days, Vietnam^47^ and Tanzania^37^ set 3 days. Vanuatu^66^ set 5 days, Thailand^80^ and Zambia,^72^ and Swaziland^55^ set 14, 14 and 35 days accordingly. Cambodia set 3^56^ or 14^60^ days, whereas Namibia set 2^68^ or 35^67^ days.
Other focus investigation and responses^9 31 32 35 40 42 44 45 48 62^	Mosquito vector monitoring Identification of mosquito breeding sites and elimination Indoor residual spraying Behavioural change communication Distribution of insecticide-treated bed nets, long-lasting insecticide-treated bed nets or long-lasting insecticide-treated hammock nets	Focus response team	Vector surveillance tools Chemicals for indoor residual spraying -Behavioural change communication materials Insecticide-treated bed nets, long-lasting insecticide-treated bed nets and/or long-lasting insecticide-treated hammock nets	Within 7 days is the standard in 1-3-7 approach

CHW, community health worker; GPS, Global Positioning System; RACD, reactive case detection.

Eleven studies in China specifically evaluated the 1-3-7 approach. In the GMS, Cambodia, Lao People Democratic Republic and Thailand follow the 1-3-7 schedule. Myanmar practically applies the 1–7 approach (case notification within 1 day, and case and focus investigations within 7 days), and Vietnam applies the 2–7 approach (case notification and investigation within 2 days and focus investigation and responses within 7 days). Among the 34 studies conducted across 10 countries in Africa, RACD was the main strategy (55.9%, 19/34 studies). Similarly, two studies in South America reported RACD, while the Columbia study also reported case notification and case investigation (online supplemental table S1).

##### Case notification

Case notification refers to the notification of a confirmed index malaria case to respective nominated health authorities usually at the commune, township or district levels. 29 RASR studies (nine in the GMS, 12 in other regions of the Asia Pacific, seven in Africa and one in South America) investigated reactive surveillance activities initiated by case notification of passively detected cases at a health facility (15 studies) or by a malaria community health worker (CHW) in the community (14 studies) (table 1). Time schedules of case notification varied but notification within 1 day after malaria diagnosis was a standard in a majority of studies,^922^,^46^ with the exception of Vietnam,^47 48^ Thailand^30^ and Indonesia,^30^ where the deadline for case notification was 2, 3 and 30 days, respectively, as per the national guidelines at the time of implementation. In studies which reported methods of notification, methods included traditional paper-based reporting,^28 34 42 48^ Short Message Services (SMS),^3643^,^45^ direct phone calling^29 36 37 42 47^ and through web-based or mobile phone-based applications.^22 23 25 28 31 48^ In almost all studies (25/29), case notification was done by front-line healthcare providers such as CHWs and health centre staff (table 1).

##### Case investigation and classification

40 studies reported case investigation (24 in the Asia Pacific, 15 in Africa and one in South America). After receiving notification from the front-line healthcare providers, responsible staff at commune, district and province levels travelled to the index case for case investigation.^922^,^25 27 29^ Two studies in Zambia re-assigned CHWs for case investigation.^44 64^ Case investigation was carried out using a set questionnaire developed by the national malaria programme. It mainly explored socio-demographic characteristics, date of onset of fever, treatment progress, travel history to trace the source of infection and to consider onward transmission, blood transfusion history, past history of malaria and treatment taken, and access/utilisation of vector control and personal protection measures.^29 33 42 44 48 63^ In some studies in Africa and the GMS, global positioning system (GPS) coordinates were also recorded to locate the index case household.^42 45 48 52 63^

During case investigation, the case was classified as per the WHO case classification (indigenous, imported, introduced, induced, relapse and recrudescent^4^) in 23 RASR studies. In the case investigation procedure in the Asia-Pacific, supervision and following up on adherence to malaria treatment, checking malaria prevention measures used by the index case and educating the index case on malaria risk factors and prevention were also included^42 48 62^ (table 1).

##### Focus investigation and responses including RACD

24 studies reported focus investigation other than RACD, while 21 studies reported focus responses (online supplemental table S1). Focus investigation included investigation of index case households and the surrounding environment. The majority of the studies (57/69) performed RACD as a form of focus investigation and response (online supplemental table S2). After notification and investigation of index cases (indigenous or imported cases) identified through passive case detection, the RACD mechanism was triggered. In RACD events implemented by almost all the studies (56/69), households of index cases were followed up to screen for malaria among household members. In 21 of these studies, surrounding households were also screened for malaria. The radius or geographical boundaries of screening (hot-spot) around the index case household varied considerably across studies, from 3 km^65^ in Brazil to 500 m in studies conducted in Africa and the Asia-Pacific.^41 42 55 66 67^ Other RACD implementation approaches included screening of four nearest households in Columbia^49^ and Africa,^68 69^ 20–30 nearest households in Vietnam^48^ to the whole village in GMS^47^ and China^23^ or the whole city in India.^40^ Two studies in Cambodia screened co-travellers or co-exposed persons (hot-pop) in their RACD implementation.^35 70^ The types of malaria diagnostic tests used to detect index and secondary cases in RACD included RDT (n=29), microscopy (n=8), polymerase chain reaction (n=19), loop-mediated isothermal amplification (n=4) or a combination of any two or three of the aforementioned tools (n=10) (table 1 and online supplemental table S2).

The common procedure of RACD included visiting the index case and surrounding households, identification of households or areas for screening of malaria through mapping via GPS, listing and ranking of households, clinical assessment of people in the households including screening of fever and other malaria signs and symptoms, assessment of vector control and personal protection measures, screening of malaria using a diagnostic test, treating or referring the secondary malaria cases identified in the screening as per the National Malaria Treatment Guidelines, and recording and reporting of the RACD events.^3742 44 45 47 48 54 57 71^,^74^ In some studies in Africa and South America, weekly mass fever and malaria screening was performed up to 16 weeks following the detection of the index case in the foci.^49 51^ In four African studies, a questionnaire was additionally administered to household members in the foci to obtain demographic information, knowledge of malaria transmission, malaria symptoms, travel history and malaria prevention methods applied.^6875^,^77^ If a majority of household members were missing during the visit, staff attempted to revisit the area in the following days.^42 48 78 79^ The RACD was usually performed in a team including a microscopist or lab technologist, surveillance officer, midwife, CHW or village leader, and the field coordinator^77 79^ (table 1 and online supplemental table S2).

In conjunction with RACD, other vector surveillance and control measures such as mosquito vector monitoring, identification of mosquito breeding sites and their elimination, indoor residual spraying, behavioural change communication, distribution of insecticide-treated bed nets or long-lasting insecticide-treated bed nets were conducted as part of the focus response mechanism across several studies performed in the Asia-Pacific^9 31 32 35 40 42 48 62^ (table 1).

### Implementation fidelity of reactive surveillance and response strategies

Implementation fidelity of RASR strategies was determined by estimated overall and geographical region-stratified completeness and timeliness of case notification, investigation and RACD.

#### Completeness and timeliness of case notification

There were significant differences in the pooled geographical region-specific levels of completeness for case notification (χ^2^(3)=47.0, p<0.001) across the geographical regions. The strata-specific completeness for China was 100% (95% CI 100% to 100%). In the GMS, overall completeness was estimated at 95% (95% CI 67% to 100%), with studies in Thailand^30^ and Vietnam^47 48^ achieving 100% completeness, while Myanmar exhibited markedly lower completeness (33%, 95% CI 30% to 36%),^34^ driving the heterogeneity in completeness estimate for the GMS. For the other Asia-Pacific countries, only one study conducted in Indonesia measured completeness, estimating it to be 93% (95% CI 87% to 97%).^30^ In Africa, the strata-specific estimate of completeness was 85% (95% CI 69% to 95%), but there was considerable heterogeneity in the completeness of case notification (I^2^=99.4%, 95% PI 18%–100%), with study-specific estimates ranging from 67% to 99%. Overall completeness was estimated to be 96% (95%CI 88% to 100%; 95% PI 44%–100%); however, heterogeneity was very high (I^2^=99.7%, χ^2^(16)=5570.5, p<0.001) (online supplemental figure S1).

**Figure 2:**
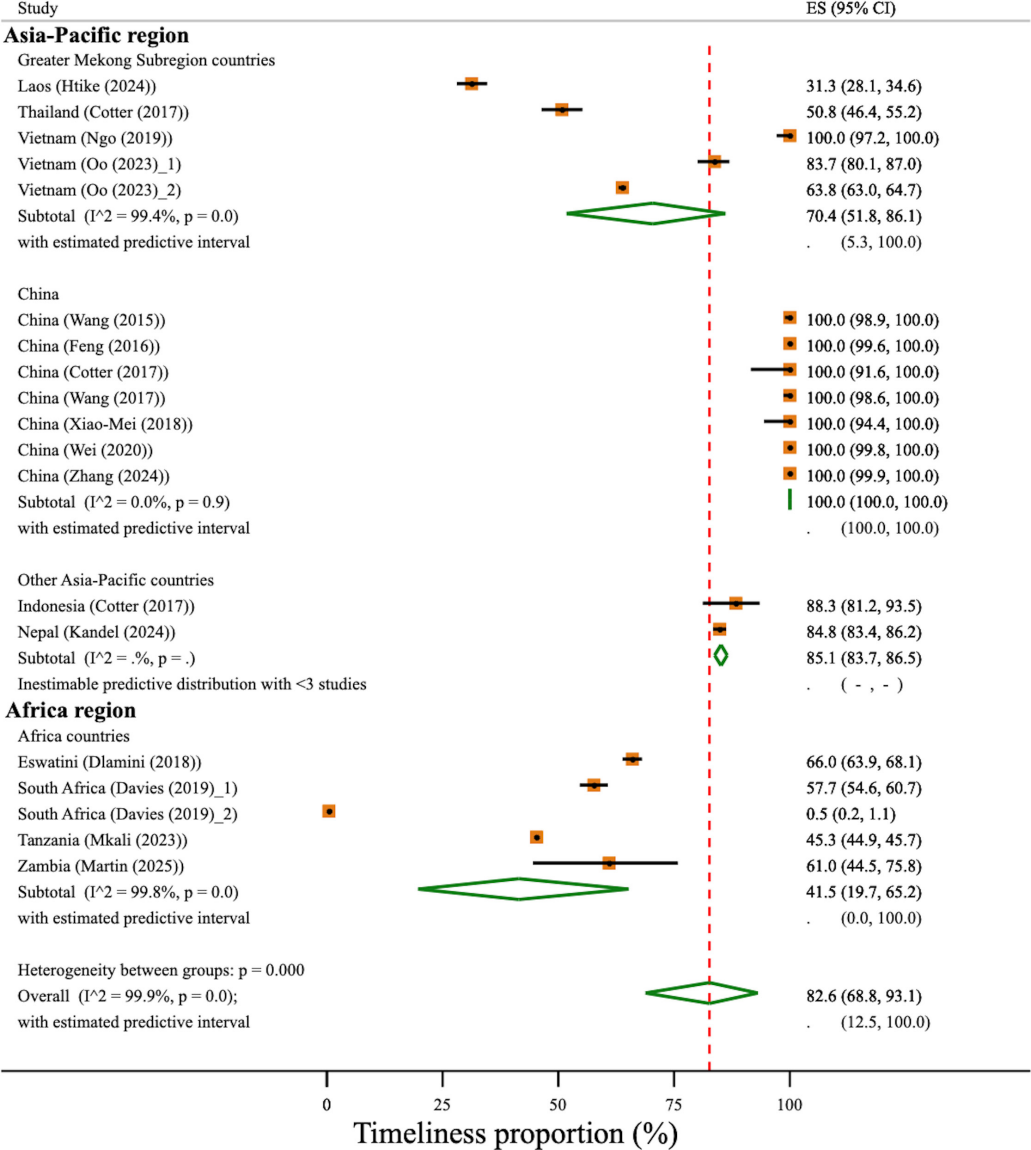
Timeliness of malaria case notification stratified by geographical region. Study estimates and pooled proportion using DerSimonian and Laird random effect with Freeman-Tukey Double Arcsine Transformation. Red-dotted line indicates the overall timeliness of case notification. Davies (2019)_1 and Oo (2023)_1, online or web-based notification; Davies (2019)_2 and Oo (2023)_2, paper-based notification. Predictive interval is inestimable for China and other Asia-Pacific region due to very large between-study variance τ2. ES, estimated proportion.

Similarly, there were significant differences in the pooled geographical region-specific levels of timeliness for case notification (χ^2^(3)=1253.6, p<0.001) across the geographical regions. All studies from China reported a 100% timeliness in case notification (95% CI 100% to 100%).^22 23 25 27 30 31 46^ In the GMS, Vietnam^47^ reported 64% to 100% timeliness in case notification depending on notification methods, but Thailand^30^ exhibited a much lower timeliness (51%, 95% CI 46% to 55%). Other Asia-Pacific regions measuring timeliness included Indonesia^30^ and Nepal^43^ studies with an estimated level of timeliness of 85% (95% CI 84% to 87%). In the Africa region, the strata-specific timeliness was estimated at 42% (95% CI 20% to 65%); however, substantial heterogeneity in the timeliness of case notification was observed with study-specific estimates ranging from 0.5% to 66%. The overall timeliness of case notification was estimated to be 83% (95% CI 69% to 93%; 95% PI 13% to 100%); however, heterogeneity was very high (I^2^=99.9%, χ^2^(18)=23021.8, p<0.001) (figure 2).

#### Completeness and timeliness of case investigation and classification

There were significant differences in the pooled geographical region-specific levels of completeness for case investigation and classification (χ^2^(3)=153, p<0.001) across the geographical regions. All studies conducted in China demonstrated a 100% completeness of case investigation and classification (95% CI 100% to 100%).^922^,^25 27 30^ In the GMS, completeness was 79% (95% CI 49% to 98%). Outside China and the GMS, only one study performed in Indonesia measured completeness, estimating it to be 78% (95% CI 70% to 86%).^30^ In Africa, the strata-specific estimate of completeness was 75% (95% CI 61% to 88%), and there was considerable heterogeneity (I^2^=99.8%; 95% PI 19% to 100%) with the rest of the study-specific estimates ranging from 35% to 83%, except for two studies from Senegal, which showed a 100% completeness.^36 51^ Overall completeness was estimated to be 88% (95% CI: 80% to 94%; 95% PI 37% to 100%); however, heterogeneity was very high (I^2^=99.9%, χ^2^(22)=17537.6, p<0.001) (online supplemental figure S2).

**Figure 3:**
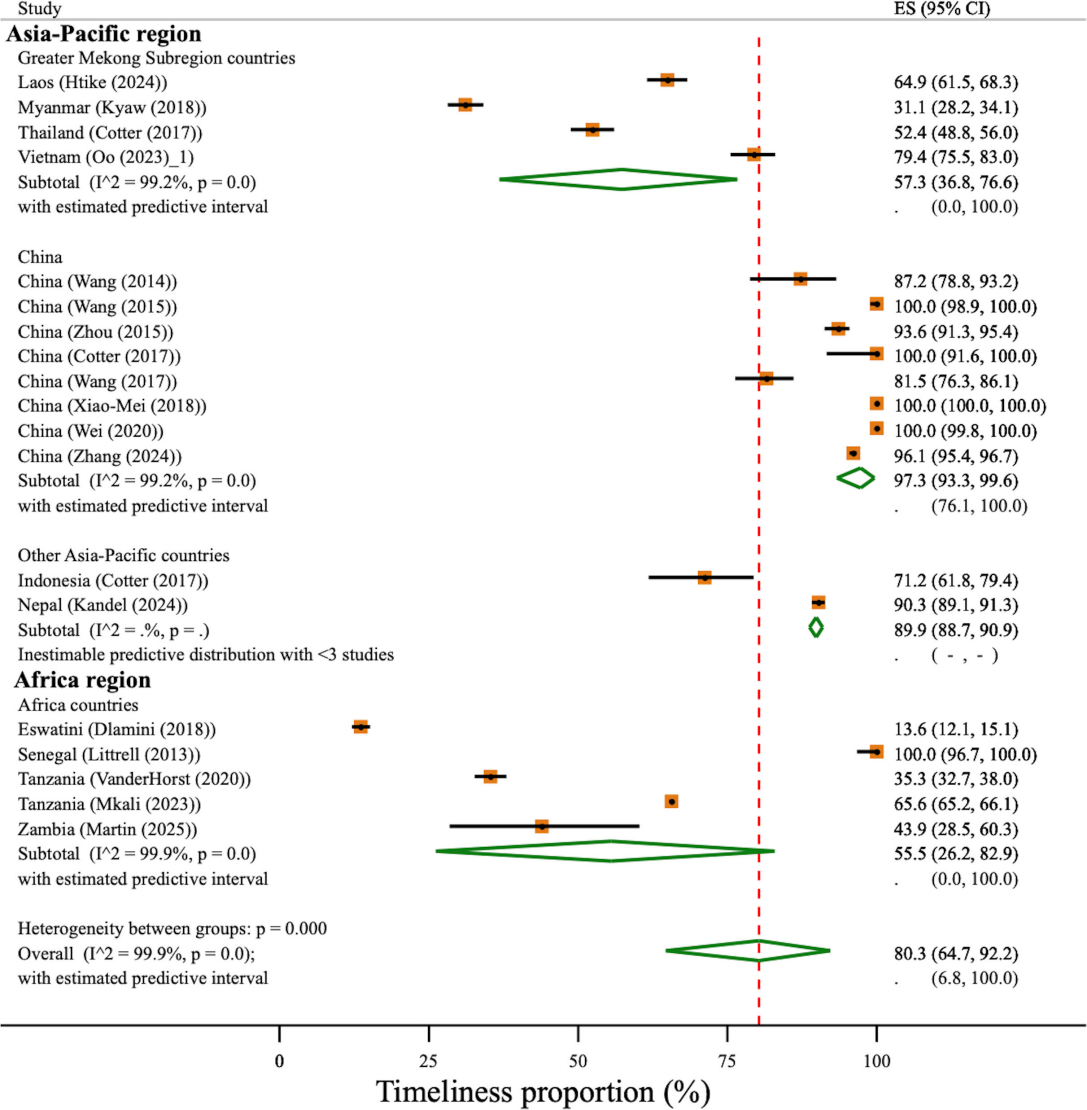
Timeliness of malaria case investigation stratified by geographical region. Study estimates and pooled proportion using DerSimonian and Laird random effect with Freeman-Tukey Double Arcsine Transformation. Red-dotted line indicates the overall timeliness of case investigation and classification. Predictive interval is inestimable for China and other Asia-Pacific region due to very large between-study variance τ2. ES, estimated proportion.

Likewise, there were significant differences in the pooled geographical region-specific levels of timeliness for case investigation and classification (χ^2^(3)=30.7, p<0.001) across the geographical regions. In China, the strata-specific estimate of timeliness was 97% (95% CI 93% to 100%), with four studies reporting 100% timeliness of case investigation and classification.^22 25 27 30^ In GMS, overall timeliness was estimated at 57% (95% CI 37% to 77%) and other Asia-Pacific countries including Indonesia^30^ and Nepal^43^ with strata-specific timeliness estimated at 90% (95% CI 89% to 91%). In Africa, the strata-specific timeliness was 56% (95% CI 26% to 83%) with considerable heterogeneity in the level of timeliness, including one study performed in Senegal which reported 100% for timeliness.^36^ Overall timeliness was estimated to be 80% (95% CI 65% to 92%, 95% PI 7% to 100%); however, heterogeneity was very high (I^2^=99.9%, χ^2^(18)=30988.5, p<0.001) (figure 3).

#### Completeness and timeliness of RACD

There was very weak evidence of significant difference in the pooled geographical region-specific levels of completeness of RACD across geographical region (χ^2^(3)=0.6, p=0.893). The strata-specific completeness of RACD for China was 80% (95% CI 8% to 100%), with one study in China exhibiting a very low level of completeness (12%, 95% CI 11% to 12%^23^), driving heterogeneity in the level of completeness compared with other study-specific estimates which were >80% (online supplemental figure S3a). Likewise, in the GMS, overall completeness was estimated at 90% (95% CI 15% to 100%) with studies in Cambodia^35^ and Thailand^80^ achieving 100% completeness. A study performed in Vietnam demonstrated substantially lower completeness (11%, 95% CI 10% to 11%^47^) and drove the heterogeneity in the level of completeness in the regional estimate (online supplemental figure S3b). The strata-specific completeness in other Asia-Pacific countries was 80% (95% CI 42% to 100%), with one study performed in Bhutan demonstrating markedly lower completeness (10%, 95% CI 10% to 11%^81^) compared with other studies performed in the region (all ≥76% (online supplemental figure S3c), contributing to observed heterogeneity in the level of completeness reported in that region. In Africa, the strata-specific completeness was estimated at 70% (95% CI 57% to 82%) with considerable heterogeneity (I^2^=100%, 95% PI 6% to 100%) in the completeness of RACD across studies (online supplemental figure S3d). Overall completeness was estimated to be at 75% (95% CI 63% to 85%; 95% PI 3% to 100%); however, heterogeneity was very high (I^2^=100%, χ^2^(41)=154846, p<0.001) (online supplemental figure S3).

**Figure 4:**
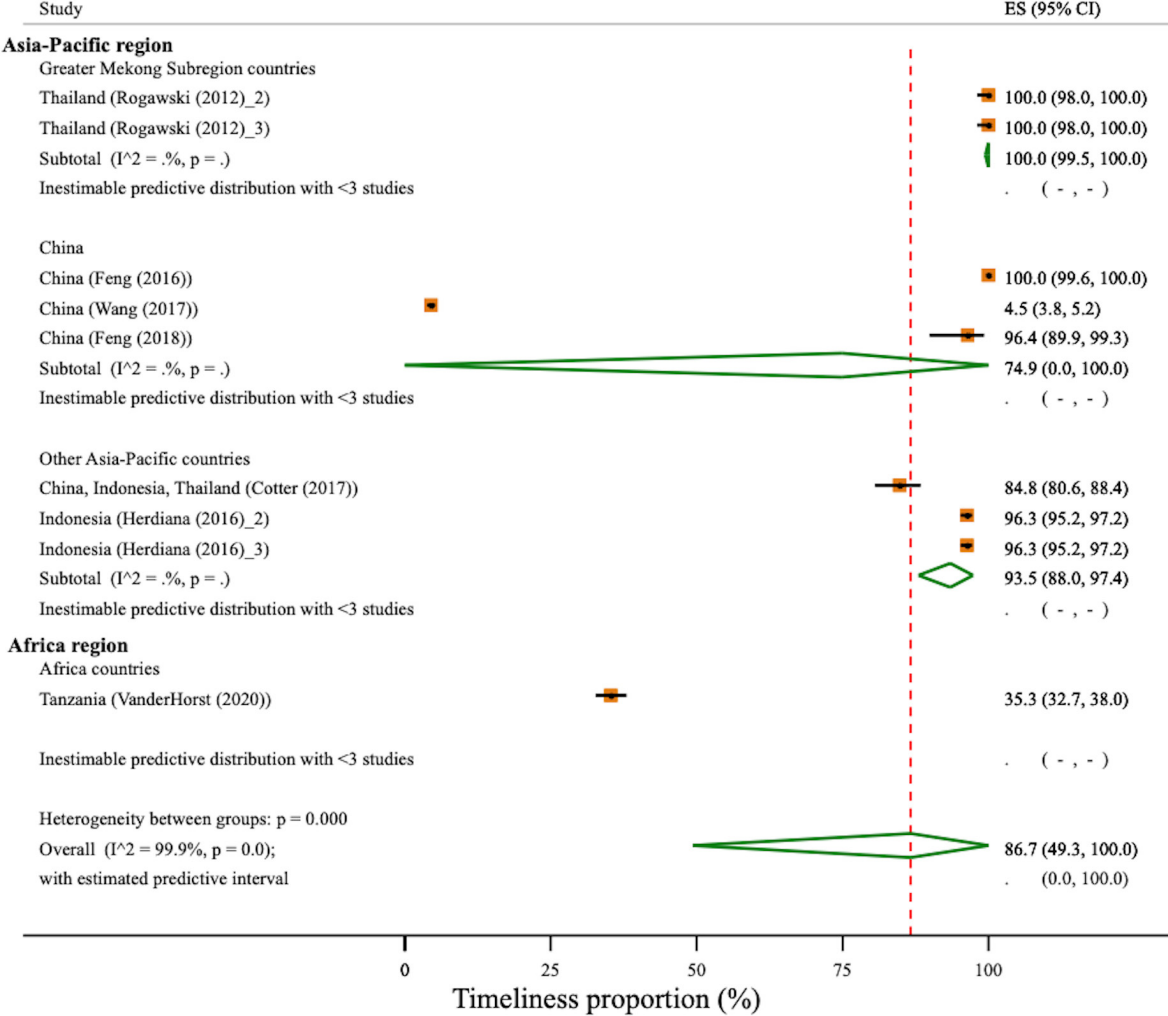
Timeliness of reactive case detection stratified by geographical region. Study estimates and pooled proportion using DerSimonian and Laird random effect with Freeman-Tukey Double Arcsine Transformation. Red-dotted line ­indicates the overall timeliness of reactive case detection (RACD). Rogawski (2012)_2 and Herdiana (2016)_2 used microscopy for RACD. Rogawski (2012)_3 and Herdiana (2016)_3 used PCR for RACD. Predictive interval is inestimable for China and other Asia-Pacific region due to very large between-study variance τ2. ES, estimated proportion.

There was a significant difference in the pooled geographical region-specific levels of timeliness of RACD across the geographical regions (χ^2^(3)=1024.2, p<0.001). The strata-specific timeliness of RACD for China was 75% (95% CI 0% to 100%) with one study reporting 4% timeliness and contributing to observed heterogeneity across studies measuring timeliness in China.^23^ The strata-specific timeliness for the GMS was 100% (95% CI 99.5% to 100%), and in other Asia-Pacific countries, timeliness was estimated to be 94% (95% CI 88% to 97%). A single African study performed in Tanzania measured timeliness and reported an estimated 35% (95% CI 33% to 38%)^37^ timeliness. Overall timeliness was estimated to be at 87% (95% CI 49% to 100%; 95% PI: 0% to 100%); however, heterogeneity was very high (I^2^=99.9%, χ^2^(8)=12124.8, p<0.001). (figure 4).

## Discussion

Understanding the implementation process and fidelity of RASR strategies across the spectrum of malaria endemic regions is essential to identify factors to improve their implementation. This review aims to provide a holistic review of the implementation and fidelity of RASR strategies. All included papers were published between 2010 and 2025, which reflects the recent use and evaluation of RASR strategies globally. There was significant methodological and statistical heterogeneity, and variations in implementation of each step of RASR across the included studies. Subgroup meta-analyses revealed significant heterogeneity among the geographical regions, except for completeness of RACD. Hence, the level of confidence in pooled estimates derived from meta-analyses is low, making formal comparison of the implementation fidelity of RASR strategies measured by completeness and timeliness of each step of RASR difficult. Nevertheless, individual study results revealed that implementation fidelity of RASR in China was maximal (100% completeness and timeliness in most studies), while studies in Myanmar and many African countries showed less than 50% completeness and timeliness of RASR strategy. Therefore, health system characteristics of an endemic country or region are critical in strengthening its surveillance system including the RASR strategies for malaria and, hence, for achieving the goal of malaria elimination nationally and regionally.

### Case notification

To trigger RASR, complete and timely case notification of the confirmed index malaria case to respective nominated health authorities is necessary. Globally, many endemic countries have already announced that malaria is a notifiable disease. It is mandatory for any healthcare providers either in public or private sector to report malaria cases to the respective authorities. In this regard, the global standard of completeness of malaria case notification should be 100%. However, this review identified that some studies have rates of completeness <80% and some as low as 33% which must be improved.

For timely notification, this review found that there was no standard deadline for reporting malaria cases globally. This might be due to the differences in health system capacity across malaria endemic regions, such as availability of electricity, coverage of mobile phone network and internet access, and capacity of frontline malaria service providers. For example, China set the deadline for case notification at 1 day because its infrastructure and health system could accommodate this requirement. However, the health system capacity and infrastructure of other GMS countries may be more limited and may explain, for example, why Thailand and Laos achieved ≤51% timely reporting in 1 day, whereas Vietnam, setting a deadline of reporting within 2 days, achieved 100% timely reporting of malaria cases. Further, we observed differences in timely notification between and also within malaria endemic countries according to the method of case notification. For instance, in Vietnam and South Africa, timeliness levels were distinct with different reporting methods where web-based online reporting outperformed the paper-based methods. The timeframe and method for malaria case notification should reflect the local health system capacity and feasibility while maintaining a reasonable timeframe for follow-up actions of case and focus investigation, classification and responses to limit onward transmission.

### Case investigation

Countries across different regions with different malaria transmission structures implement case investigation in different ways. Studies in China demonstrated 100% completeness and higher level of timeliness (97%) in case investigation, while studies in the GMS and Africa had low completeness (≤79%) and timeliness (≤57%). In some of these GMS and African studies, the low percentages of completeness and timeliness may be due to the fact that case investigation as a step of RASR strategy was introduced early while there were still many malaria cases.^2^ In the control phase where there are many malaria cases, not all notified malaria cases may be investigated given the limitations of health system capacity and usefulness of doing so. Frontline malaria providers could not perform case investigation and classification to all notified malaria cases. In reality, frontline malaria providers often have many roles and responsibilities at the commune and district levels. They are working for the control and elimination of many communicable diseases as well as for the improvement of maternal and child health. Therefore, case investigation should be prioritised only when an area has achieved a low annual parasite incidence (typically less than one per 1000 at-risk population according to the WHO)^3^ and the national health system is ready to do so. In the context of limited health system capacity in the formal health sector, an alternative approach would be task shifting of case investigation and classification to CHWs as field tested in Myanmar^82^ and Cambodia.^35^

### Foci investigation and responses including RACD

Foci investigation is essential to contain and address any possible malaria cluster associated with the index case. RACD itself is also diverse in terms of the radius of screening, diagnostic methods and time schedule. For instance, a recent review^83^ recommended neighbours should be inclusive in the RACD based on their risks of malaria infection. Furthermore, there is no consensus on how focus investigation and responses should be performed globally. Studies in Africa mainly use RACD, while the included studies in China and the Asia-Pacific, including the GMS, mainly implement RACD as well as other focus responses such as vector surveillance and control. There are other focus response activities such as reactive drug administration and reactive indoor residual spraying that could be implemented as part of focus response depending on the type of malaria focus (active, residual non-active or clear).

This systematic review demonstrated that the proportion of RACD, which is complete and undertaken in a timely manner, varies among studies implemented across the diverse geographical regions. This may be due to the recent introduction and adoption of RASR strategies but without much available regional and global evidence. Another systematic review^13^ also described RACD timeliness varied across geographical locations where topographic and demographic factors pose a barrier to that. Countries approaching malaria elimination and implementing RACD in the elimination programme should consider adapting existing RACD strategy with updated regional and global evidence in order not to miss any secondary malaria cases associated with the index case but within the allowance of national health system capacity.

To ensure completeness of the RASR strategy, countries in Africa may consider adopting the 1-3-7 where there is sufficient capacity within the local health infrastructure. This is supported by a recent review, which illustrates the success of 1-3-7 implementation fidelity when tailored and adapted based on the local context.^84^ Further, it was recently demonstrated that timeliness of each RASR component in countries other than China varies considerably and is highly dependent on availability of human and financial resources, infrastructure and consistent compliance where transnational borders mainly impose a challenge.^85^

### Strengths and limitations

This paper is a comprehensive systematic review with meta-analysis that explores all three key steps of the RASR strategy: case notification, case investigation and classification, and focus investigation and responses including RACD. It had no restriction in study design of the included studies used to explore RASR strategies, type of document published and language of publication, and included all studies in malaria endemic regions.

Although no subgroup analysis was planned in the review protocol, meta-analyses to obtain overall and geographical region-specific pooled estimates were performed. There was significant statistical heterogeneity both within and across geographical regions, which reduced confidence in pooled estimation of levels across the key implementation fidelity outcomes of the RASR. This was most likely due to heterogeneity in study methods and implementation of RASR such as methods and time schedule set for each RASR step. However, exploring individual contextual or health system-related factors associated with the implementation fidelity of each step of RASR was beyond the scope of this review. Heterogeneity within studies may arise due to diversity in health infrastructure and health system readiness between regions of the same country. Moreover, in instances where multiple outcome data are extracted from the same study, meta-analyses may underestimate standard errors. Importantly, the outcomes of interest included in this study only assess implementation process and fidelity of RASR rather than the effectiveness of RASR strategies on preventing onward transmission of malaria, which is influenced by multiple factors that were not assessed.

## Conclusion

Overall, malaria-endemic countries in the Asia-Pacific are adopting the 1-3-7 RASR strategy, while many African countries implement RACD. Implementation fidelity of the RASR strategy varies within and across countries and regions. This may be due to differences in regional, national and sub-national health system capacity and infrastructure. It is recommended that each endemic country implementing the malaria elimination programme should tailor 1-3-7 strategies according to national health system capacity considering the global and regional evidence.

## Supplementary material

10.1136/bmjph-2024-001180 online supplemental file 1

10.1136/bmjph-2024-001180 online supplemental file 2

10.1136/bmjph-2024-001180 online supplemental file 3

10.1136/bmjph-2024-001180 online supplemental file 4

10.1136/bmjph-2024-001180 online supplemental file 5

10.1136/bmjph-2024-001180 online supplemental file 6

## Data Availability

All data relevant to the study are included in the article or uploaded as supplementary information.
