# Supplementary material for: Implementation and fidelity of reactive surveillance and response strategies for malaria elimination: a systematic review and meta-analysis
Source: BMJ Public Health. 2025 Nov 13;3(2):e001180. doi: 10.1136/bmjph-2024-001180 (PMC12625913; doi:10.1136/bmjph-2024-001180)
Supplement: online supplemental file 1 [file bmjph-3-2-s001.pdf]

Supplementary material 1 – Systematic review protocol

To enable PROSPERO to focus on COVID-19 submissions, this registration record has undergone basic automated checks for eligibility and is published exactly as submitted. PROSPERO has never provided peer review, and usual checking by the PROSPERO team does not endorse content. Therefore, automatically published records should be treated as any other PROSPERO registration. Further detail is provided here.

Citation

Julia Cutts, . Kaung Myat Thu, . Win Han Oo, . Win Htike, Freya Fowkes, Paul Agius, . Galau Naw Hkawng, Katherine O'Flaherty, . May Chan Oo. Acceptability, feasibility and effectiveness of malaria reactive surveillance and response strategies: A systematic review. PROSPERO 2021 CRD42021249857 Available from: https://www.crd.york.ac.uk/prospero/display_record.php?ID=CRD42021249857

Review question

1) What malaria reactive surveillance and response strategies are being implemented in malaria endemic countries, particularly those approaching malaria elimination?

2) What is the effectiveness of different malaria reactive surveillance and response strategies in detecting malaria cases, reducing malaria burden, and advancing malaria elimination goals in malaria endemic countries, particularly those approaching malaria elimination?

3) What is the acceptability of malaria reactive surveillance and response strategies according to community members, local authorities and health stakeholders in malaria endemic countries?

4) What is the feasibility of implementing reactive surveillance and response strategies in malaria endemic countries?

5) What are the barriers and enablers to the successful implementation of reactive surveillance and response strategies?

Searches

For published studies, online electronic databases namely PubMed, Web of Science, Scopus, African Index Medicus, and LILACS (Latin American and Caribbean Health Sciences Literature) will be searched for quantitative and qualitative studies published in all years up to current date. The reference lists of included studies will be searched for additional studies. Studies and relevant documents reported in languages other than English will be included and translated into English by translators, or where native speakers are unavailable, by using online translation applications. Relevant white and grey literature relating to reactive surveillance and response strategies will be sourced from websites such as the following:

•UN agencies (World Health Organization http://www.who.int/en/ , UNICEF http://www.unicef.org/ , UNOPS https://www.unops.org/english/Pages/Home.aspx);
• International Organizations (IO) and Non-Government Organizations (NGO) (Population Services International http://www.psi.org/ , Malaria Consortium http://www.malariaconsortium.org/

• International Organizations for Migration http://www.iom.int/ , Save the Children International https://www.savethechildren.net/ , Médecins Sans Frontières (MSF) International http://www.msf.org/ , Community Partners International http://cpintl.org/);

• Philanthropies and donor agencies (Bill and Malinda Gate Foundation http://www.gatesfoundation.org/ , United States Agency for International Development https://www.usaid.gov/ , UK Department for International Development https://www.gov.uk/government/organisations/department-for-international-development ,

• Australian Department of Foreign Affairs and Trade http://dfat.gov.au/pages/default.aspx , Asia Development Bank http://www.adb.org/ , Japan International Cooperation Agency http://www.jica.go.jp/english/).

• [OpenGrey (www.opengrey.eu; to date of search)]

• [Agency for Healthcare Research and Quality (AHRQ; www.ahrq.gov; to date of search)]

• [National Institute for Health and Clinical Excellence (NICE; www.nice.org.uk; to date of search).]

• EThOs

First search conducted 20 January 2021.

Search strategy

https://www.crd.york.ac.uk/PROSPEROFILES/249857_STRATEGY_20210615.pdf

Types of study to be included

Inclusions:

Primary qualitative, quantitative, and mixed methods studies will be eligible for inclusion in this review.

Quantitative and mixed-methods studies will include population-based cross-sectional, case-control, cohort studies, randomized controlled trials (RCTs), mixed methods studies, program evaluations, and feasibility studies. Systematic reviews with similar research aims will be identified and discussed where appropriate. Studies that employ qualitative study designs such as ethnography, phenomenology, case studies, grounded theory studies and qualitative process evaluations will be considered for inclusion. Studies that use both qualitative methods for data collection (e.g. focus group discussions, interviews, observation, document analysis, open-ended survey questions) and qualitative methods for data analysis (e.g. thematic analysis, framework analysis, grounded theory) will be considered for inclusion. Qualitative studies from grey literature are also eligible.

Exclusions

• Case reports or case series

• Letters, editorials, commentaries, reviews or other articles that do not present primary findings or data.

• Study protocols

• Conference abstracts

Condition or domain being studied

This review will examine malaria, an infectious disease caused by *Plasmodium* spp. parasites transmitted by *Anopheles* mosquitoes. Globally, malaria causes over 200 million cases per year and over 400, 000 deaths.

Participants/population

Study populations will include those living in areas at risk of malaria (stable areas where Plasmodium Falciparum Annual Parasite Index (PfAPI) ?0.1 per 1, 000 pa & unstable areas where PfAPI > 0 and < 0.1 per 1, 000 pa) as defined by the Malaria Atlas Project [24]. In addition, health care and key groups/individuals who have direct or indirect interactions with populations/individuals living in areas at risk of malaria will also be included where relevant. Examples include village heads, ministers of health, malaria program leads, malaria patients and other community members at risk of malaria, and public health officials.

Intervention(s), exposure(s)

This review will focus on malaria prevention, control and elimination activities undertaken in direct response to the detection of a positive malaria case, described herein as “reactive surveillance and response interventions”. In some contexts these activities are collectively referred to as “Case Investigation, Foci Investigation and Response, CIFIR”. All reactive surveillance and response interventions will be included in the review, including overarching time-bound policies such as the 1-3-7 approach, as well as specific focus response activities like reactive surveillance, prevention, and control activities conducted in response to the identification of an initial index case or foci. For example, reactive case detection, supplementary vector control, and community awareness activities.

Comparator(s)/control

The comparator will be as defined in individual studies, but in most cases is likely to be the absence of the intervention (i.e. non-exposed control group).

Context

This review will include studies assessing the acceptability, feasibility and effectiveness of reactive surveillance and response strategies for malaria in all/any malaria endemic areas worldwide.

Main outcome(s)

The primary criterion for study inclusion is examination of the acceptability, feasibility, or effectiveness of a malaria reactive surveillance and response strategy or strategies in a malaria endemic area. Outcomes relating to the effectiveness of reactive surveillance and response will include but will not be limited to, the number of positive malaria cases identified by microscopy, RDT, or PCR (or any combination thereof), the number of people screened for malaria symptoms, the number of malaria tests performed at either a local or national level. For example, number of cases identified by reactive case detection, foci investigations, and other response activities precipitated by the identification of an initial index case of malaria. This review will consider the perceptions and experiences of health care workers, policy makers, and other malaria program stakeholders, malaria patients and other community members with particular attention paid to findings relating to the acceptability, feasibility as well as barriers and enablers to the implementation of reactive surveillance and responses interventions in malaria endemic countries.

Measures of effect

Quantitative studies

Narrative descriptions of study characteristics, outcome measures, and key findings will be reported. For estimates of the effectiveness of reactive surveillance and response strategies on malaria testing and cases numbers, measures of association (odds ratio (OR), risk ratio (RRs), incidence rate ratio (IRR), or hazard ratios (HR)) and their 95% confidence intervals (CIs) will be extracted or derived using data reported in the publications. Where a study did not provide measures of association the study results will be included in narrative terms only.

Qualitative studies

Malaria reactive surveillance and response strategies currently worldwide will be described in narrative terms, using information extracted from published studies and white and grey literature. Qualitative data will be summarized as a comparative content analysis facilitated by matrix displays. Narrative reporting of qualitative data synthesis will be structured around SURE guideline categories (SURE Collaboration, 2011).

Additional outcome(s)

Not applicable

Data extraction (selection and coding)

One investigator will conduct initial literature searches and screen titles and abstracts for inclusion. Two investigators will independently screen the full text of identified studies against inclusion and exclusion

criteria, with discrepancies resolved by discussion with a third investigator. Covidence will be used as a tool to coordinate the screening process.

Two investigators will independently undertake data extraction using a data extraction form (Appendix X). Discrepancies will be resolved following discussion with a third investigator. The data extraction form will cover the following information:

• Basic study/publication information (e.g. first author, journal/source, date of publication)

• Basic characteristics of study (e.g. study site, study design, participants, objectives)

• Details of reactive surveillance and response intervention(s) being studied

• Outcomes including acceptability, case detection, feasibility, barriers and enablers.

• Qualitative findings: themes, findings and quotations

• Quantitative findings: data source, outcome measures, summary of results.

• Conclusions

Risk of bias (quality) assessment

For studies presenting quantitative findings, The Risk of Bias In Non-randomized Studies – of Interventions (ROBINS-I) assessment tool will be used to assess the risk of bias and classify studies in one of five levels: low risk, moderate risk, serious risk and critical risks of bias, and no information. Quality and risk of bias in studies presenting qualitative data will be assessed using CERQual https://methods.cochrane.org/qi/news/cerqual-new-approach-supporting-use-qualitative-evidence-decision-making and/or CASP tools https://casp-uk.net/casp-tools-checklists/.

Strategy for data synthesis

Quantitative studies

Narrative descriptions of study characteristics, outcome measures, and key findings will be reported. For estimates of the effectiveness of reactive surveillance and response strategies on malaria testing and cases numbers, measures of association (odds ratio (OR), risk ratio (RRs), incidence rate ratio (IRR), or hazard ratios (HR)) and their 95% confidence intervals (CIs) will be extracted or derived using data reported in the publications. Where there are two or more studies that can be combined (e.g. same intervention and same outcome), a pooled estimate for each outcome will be calculated using a random-effects model. Where a study did not provide measures of association for the association between reactive surveillance and responses interventions and effectiveness in reducing malaria cases, or increasing screening or testing of potential malaria cases (or they could not be calculated using the information provided) the study results will be included in narrative terms only. Quantitative data will be summarized as a descriptive analysis (and inferential analysis whenever possible) using frequencies, distributions, ratios, means and proportions. Dichotomous outcomes will be presented using proportions with 95% confidence interval (CI). Continuous outcomes (number and/or percentage of people diagnosed with malaria by RDT, microscopy or PCR, number and/or percentage of people who died from malaria, quantitative measures of barriers and enablers) will be analyzed using difference in means (with 95% CI) or medians.

Qualitative studies

Malaria reactive surveillance and response strategies currently worldwide will be described in narrative terms, using information extracted from published studies and white and grey literature. Qualitative data will be summarized as a comparative content analysis facilitated by matrix displays. Narrative reporting of qualitative data synthesis will be structured around SURE guideline categories (SURE Collaboration, 2011).

Analysis of subgroups or subsets

No subgroup analysis planned at this stage.

Contact details for further information

Julia Cutts
julia.cutts@burnet.edu.au

Organisational affiliation of the review

Burnet Institute
www.burnet.edu.au

Review team members and their organisational affiliations

Dr Julia Cutts. Burnet Institute
Dr . Kaung Myat Thu. Burnet Institute
Dr . Win Han Oo. Burnet Institute
Dr . Win Htike. Burnet Institute
Professor Freya Fowkes. Burnet Institute
Mr Paul Agius. Burnet Institute
Dr . Galau Naw Hkawng. Burnet Insitute
Dr Katherine O'Flaherty. Burnet Institute
Dr . May Chan Oo. Burnet Institute

Type and method of review

Epidemiologic, Intervention, Narrative synthesis, Systematic review

Anticipated or actual start date

15 January 2021

Anticipated completion date

15 December 2021

Funding sources/sponsors

The Global Fund to fight Aids, Tuberculosis and Malaria (GFATM)/UNOPS; Burnet Institute

Grant number(s)

State the funder, grant or award number and the date of award

GFATM/UNOPS: QSE-M-UNOPS-BI-20864-007-61; 1 January 2021

Conflicts of interest

Language

English

Country

Australia, Myanmar (Burma)

Stage of review

Review Ongoing

Subject index terms status

Subject indexing assigned by CRD

Subject index terms

MeSH headings have not been applied to this record

Date of registration in PROSPERO

16 July 2021

Date of first submission

15 June 2021

Stage of review at time of this submission

Stage	Started	Completed
Preliminary searches	Yes	No
Piloting of the study selection process	Yes	No
Formal screening of search results against eligibility criteria	Yes	No
Data extraction	No	No
Risk of bias (quality) assessment	No	No
Data analysis	No	No

The record owner confirms that the information they have supplied for this submission is accurate and complete and they understand that deliberate provision of inaccurate information or omission of data may be construed as scientific misconduct.
The record owner confirms that they will update the status of the review when it is completed and will add publication details in due course.

Versions

16 July 2021
16 July 2021
