# Supplementary material for: Implementation and fidelity of reactive surveillance and response strategies for malaria elimination: a systematic review and meta-analysis
Source: BMJ Public Health. 2025 Nov 13;3(2):e001180. doi: 10.1136/bmjph-2024-001180 (PMC12625913; doi:10.1136/bmjph-2024-001180)
Supplement: online supplemental file 2 [file bmjph-3-2-s002.pdf]

Supplementary Material 2: Excluded papers after full text screening with reasons

Study protocols

1. Bousema, T., Stevenson, J., Baidjoe, A., Stresman, G., Griffin, J. T., Kleinschmidt, I., . . . Cox, J. (2013). The impact of hotspot-targeted interventions on malaria transmission: study protocol for a cluster-randomized controlled trial. *Trials, 14*, 36. doi:10.1186/1745-6215-14-36
2. Bridges, D. J., Miller, J. M., Chalwe, V., Moonga, H., Hamainza, B., Steketee, R., . . . Larsen, D. A. (2017). Community-led Responses for Elimination (CoRE): a study protocol for a community randomized controlled trial assessing the effectiveness of community-level, reactive focal drug administration for reducing Plasmodium falciparum infection prevalence and incidence in Southern Province, Zambia. *Trials, 18*(1), 511. doi:10.1186/s13063-017-2249-0
3. Lover, A. A., Dantzer, E., Hocini, S., Estera, R., Rerolle, F., Smith, J. L., . . . Bennett, A. (2019). Study protocol for a cluster-randomized split-plot design trial to assess the effectiveness of targeted active malaria case detection among high-risk populations in Southern Lao PDR (the AcME-Lao study). *Gates Open Res, 3*, 1730. doi:10.12688/gatesopenres.13088.1
4. Medzihradsky, O. F., Kleinschmidt, I., Mumbengegwi, D., Roberts, K. W., McCreesh, P., Dufour, M.-S. K., . . . Hsiang, M. S. (2018). Study protocol for a cluster randomised controlled factorial design trial to assess the effectiveness and feasibility of reactive focal mass drug administration and vector control to reduce malaria transmission in the low endemic setting of Namibia. *BMJ Open, 8*(1), e019294. doi:10.1136/bmjopen-2017-019294
5. Wang, D., Chaki, P., Mlacha, Y., Gavana, T., Michael, M. G., Khatibu, R., . . . Zhou, X. N. (2019). Application of community-based and integrated strategy to reduce malaria disease burden in southern Tanzania: the study protocol of China-UK-Tanzania pilot project on malaria control. *Infect Dis Poverty, 8*(1), 4. doi:10.1186/s40249-018-0507-3
6. Whidden, C., Treleaven, E., Liu, J., Padian, N., Poudiougou, B., Bautista-Arredondo, S., . . . Kayentao, K. (2019). Proactive community case management and child survival:

protocol for a cluster randomised controlled trial. *BMJ Open, 9*(8), e027487. doi:10.1136/bmjopen-2018-027487

Systematic reviews and reviews

1. Canavati, S. E., Lawford, H. L., Fatunmbi, B. S., Lek, D., Top-Samphor, N., Leang, R., . . . Kazadi, W. M. (2016). Establishing research priorities for malaria elimination in the context of the emergency response to artemisinin resistance framework-the Cambodian approach. *Malar J, 15*, 120. doi:10.1186/s12936-016-1117-9
2. Cao, J., Sturrock, H. J., Cotter, C., Zhou, S., Zhou, H., Liu, Y., . . . Gao, Q. (2014). Communicating and monitoring surveillance and response activities for malaria elimination: China's "1-3-7" strategy. *PLoS Med, 11*(5), e1001642. doi:10.1371/journal.pmed.1001642
3. Cao, J., Sturrock, H. J., Cotter, C., Zhou, S., Zhou, H., Liu, Y., . . . Gao, Q. (2014). Communicating and monitoring surveillance and response activities for malaria elimination: China's "1-3-7" strategy. *PLoS Med, 11*(5), e1001642. doi:10.1371/journal.pmed.1001642
4. Dondorp, A. M., Smithuis, F. M., Woodrow, C., & Seidlein, L. V. (2017). How to Contain Artemisinin- and Multidrug-Resistant Falciparum Malaria. *Trends Parasitol, 33*(5), 353-363. doi:10.1016/j.pt.2017.01.004
5. Perera, R., Caldera, A., & Wickremasinghe, A. R. (2020). Reactive Case Detection (RACD) and foci investigation strategies in malaria control and elimination: a review. *Malar J, 19*(1), 401. doi:10.1186/s12936-020-03478-0
6. Stresman, G., Whittaker, C., Slater, H. C., Bousema, T., & Cook, J. (2020). Quantifying Plasmodium falciparum infections clustering within households to inform household-based intervention strategies for malaria control programs: An observational study and meta-analysis from 41 malaria-endemic countries. *PLoS Med, 17*(10), e1003370. doi:10.1371/journal.pmed.1003370
7. Sturrock, H. J., Hsiang, M. S., Cohen, J. M., Smith, D. L., Greenhouse, B., Bousema, T., & Gosling, R. D. (2013). Targeting asymptomatic malaria infections: active surveillance in control and elimination. *PLoS Med, 10*(6), e1001467. doi:10.1371/journal.pmed.1001467

8. van Eijk, A. M., Ramanathapuram, L., Sutton, P. L., Kanagaraj, D., Sri Lakshmi Priya, G., Ravishankaran, S., . . . Eapen, A. (2016). What is the value of reactive case detection in malaria control? A case-study in India and a systematic review. *Malar J, 15*, 67. doi:10.1186/s12936-016-1120-1
9. Yang, W. Z., & Zhou, X. N. (2016). [New challenges of malaria elimination in China]. *Zhonghua Yu Fang Yi Xue Za Zhi, 50*(4), 289-291. doi:10.3760/cma.j.issn.0253-9624.2016.04.001
10. Yi B, Zhang L, Yin J, Zhou S, Xia Z. 1-3-7 surveillance and response approach in malaria elimination: China's practice and global adaptions. Malar J. 2023;22(1):152.
11. Yi B, Zhang L, Yin J, Zhou S, Xia Z. Correction: 1-3-7 surveillance and response approach in malaria elimination: China's practice and global adaptions. Malar J. 2023;22(1):179.
12. Saeung M, Jupatanakul N, Hii J, Thanispong K, Chareonviriyaphap T, Manguin S. Overview of national and local efforts to eliminate malaria in Thailand. Trends Parasitol. 2025;41(1):52-65.
13. Steinhardt LC, Kc A, Tiffany A, et al. Reactive Case Detection and Treatment and Reactive Drug Administration for Reducing Malaria Transmission: A Systematic Review and Meta-Analysis. Am J Trop Med Hyg. 2024;110(4_Suppl):82-93.
14. Aidoo EK, Aboagye FT, Botchway FA, et al. Reactive Case Detection Strategy for Malaria Control and Elimination: A 12 Year Systematic Review and Meta-Analysis from 25 Malaria-Endemic Countries. Tropical Medicine and Infectious Disease. 2023;8(3).
15. Win H, Nay Yi Yi L, Kyawt Mon W, et al. Reactive surveillance and response strategies for malaria elimination in Myanmar: a literature review. Malar J. 2023;22(1):140.
16. Sogandji N, Stevenson A, Luo MY, Qi G, Maude RJ. Systematic review of evidence for the impact and effectiveness of the 1-3-7 strategy for malaria elimination. Malar J. 2024;23(1):371.

Wrong interventions

1. Update of: Kheang et al., Malaria Case Detection Among Mobile Populations and Migrant Workers in Myanmar: Comparison of 3 Service Delivery Approaches. (2018). *Glob Health Sci Pract, 6*(3), 612. doi:10.9745/ghsp-d-18-00358
2. Bousema, T., Stresman, G., Baidjoe, A. Y., Bradley, J., Knight, P., Stone, W., . . . Cox, J. (2016). The Impact of Hotspot-Targeted Interventions on Malaria Transmission in Rachuonyo South District in the Western Kenyan Highlands: A Cluster-Randomized Controlled Trial. *PLoS Med, 13*(4), e1001993. doi:10.1371/journal.pmed.1001993
3. Branch, O., Casapia, W. M., Gamboa, D. V., Hernandez, J. N., Alava, F. F., Roncal, N., . . . Gotuzzo, E. (2005). Clustered local transmission and asymptomatic Plasmodium falciparum and Plasmodium vivax malaria infections in a recently emerged, hypoendemic Peruvian Amazon community. *Malar J, 4*, 27. doi:10.1186/1475-2875-4-27
4. Burton, R. A., Chévez, J. E. R., Sauerbrey, M., Guinovart, C., Hartley, A., Kirkwood, G., . . . Campbell, C. C. K. (2018). Factors Associated with the Rapid and Durable Decline in Malaria Incidence in El Salvador, 1980-2017. *Am J Trop Med Hyg, 99*(1), 33-42. doi:10.4269/ajtmh.17-0629
5. Cao, Y., Cotter, C., Wang, W., Liu, Y., Zhou, H., Zhu, G., & Cao, J. (2020). Malaria Elimination in China: Improving County-Level Malaria Personnel Knowledge of the 1-3-7 Strategy through Tabletop Exercises. *Am J Trop Med Hyg, 102*(4), 804-810. doi:10.4269/ajtmh.19-0560
6. Cao, Y. Y., Zhou, H. Y., Zhu, G. D., Wang, W. M., & Cao, J. (2017). [Survey of knowledge of basic centers for disease control and prevention staffs on the "1-3-7" strategy for malaria elimination in Jiangsu Province]. *Zhonghua Yu Fang Yi Xue Za Zhi, 51*(4), 361-363. doi:10.3760/cma.j.issn.0253-9624.2017.04.016
7. Crowell, V., Hardy, D., Briët, O., Chitnis, N., Maire, N., & Smith, T. (2012). Can we depend on case management to prevent re-establishment of P. falciparum malaria, after local interruption of transmission? *Epidemics, 4*(1), 1-8. doi:10.1016/j.epidem.2011.10.003
8. Desai, M. R., Samuels, A. M., Odongo, W., Williamson, J., Odero, N. A., Otieno, K., . . . Lindblade, K. A. (2020). Impact of Intermittent Mass Testing and Treatment on

Incidence of Malaria Infection in a High Transmission Area of Western Kenya. *Am J Trop Med Hyg, 103*(1), 369-377. doi:10.4269/ajtmh.19-0735

9. Ekawati, L. L., Johnson, K. C., Jacobson, J. O., Cueto, C. A., Zarlinda, I., Elyazar, I. R. F., . . . Bennett, A. (2020). Defining malaria risks among forest workers in Aceh, Indonesia: a formative assessment. *Malar J, 19*(1), 441. doi:10.1186/s12936-020-03511-2
10. Erhart, A., Thang, N. D., Xa, N. X., Thieu, N. Q., Hung, L. X., Hung, N. Q., . . . D'Alessandro, U. (2007). Accuracy of the health information system on malaria surveillance in Vietnam. *Trans R Soc Trop Med Hyg, 101*(3), 216-225. doi:10.1016/j.trstmh.2006.07.003
11. Gaye, S., Kibler, J., Ndiaye, J. L., Diouf, M. B., Linn, A., Gueye, A. B., . . . Thwing, J. (2020). Proactive community case management in Senegal 2014-2016: a case study in maximizing the impact of community case management of malaria. *Malar J, 19*(1), 166. doi:10.1186/s12936-020-03238-0
12. Hamainza, B., Moonga, H., Sikaala, C. H., Kamuliwo, M., Bennett, A., Eisele, T. P., . . . Killeen, G. F. (2014). Monitoring, characterization and control of chronic, symptomatic malaria infections in rural Zambia through monthly household visits by paid community health workers. *Malar J, 13*, 128. doi:10.1186/1475-2875-13-128
13. Herdiana, H., Fuad, A., Asih, P. B., Zubaedah, S., Arisanti, R. R., Syafruddin, D., . . . Hawley, W. A. (2013). Progress towards malaria elimination in Sabang Municipality, Aceh, Indonesia. *Malar J, 12*, 42. doi:10.1186/1475-2875-12-42
14. Jaiteh, F., Okebe, J., Masunaga, Y., D'Alessandro, U., Achan, J., Gryseels, C., . . . Grietens, K. P. (2021). Understanding adherence to reactive treatment of asymptomatic malaria infections in The Gambia. *Sci Rep, 11*(1), 1746. doi:10.1038/s41598-021-81468-1
15. Kaehler, N., Adhikari, B., Cheah, P. Y., von Seidlein, L., Day, N. P. J., Paris, D. H., . . . Pell, C. (2019). Prospects and strategies for malaria elimination in the Greater Mekong Sub-region: a qualitative study. *Malar J, 18*(1), 203. doi:10.1186/s12936-019-2835-6
16. Kamanga, A., Moono, P., Stresman, G., Mharakurwa, S., & Shiff, C. (2010). Rural health centres, communities and malaria case detection in Zambia using mobile telephones: a

means to detect potential reservoirs of infection in unstable transmission conditions. *Malar J, 9*, 96. doi:10.1186/1475-2875-9-96

17. Kheang, S. T., Lin, M. A., Lwin, S., Naing, Y. H., Yarzar, P., Kak, N., & Price, T. (2018). Malaria Case Detection Among Mobile Populations and Migrant Workers in Myanmar: Comparison of 3 Service Delivery Approaches. *Glob Health Sci Pract, 6*(2), 384-389. doi:10.9745/ghsp-d-17-00318
18. Kondrashin, A. V., Sharipov, A. S., Kadamov, D. S., Karimov, S. S., Gasimov, E., Baranova, A. M., . . . Morozov, E. N. (2017). Elimination of Plasmodium falciparum malaria in Tajikistan. *Malar J, 16*(1), 226. doi:10.1186/s12936-017-1861-5
19. Larson, B. A., Ngoma, T., Silumbe, K., Rutagwera, M. R., Hamainza, B., Winters, A. M., . . . Scott, C. A. (2016). A framework for evaluating the costs of malaria elimination interventions: an application to reactive case detection in Southern Province of Zambia, 2014. *Malar J, 15*(1), 408. doi:10.1186/s12936-016-1457-5
20. Li, S., Yin, S., Wang, J., Li, X., & Feng, J. (2016). Shifting from control to elimination: analysis of malaria epidemiological characteristics in Tengchong County around China-Myanmar border, 2005-2014. *Malar J, 15*, 45. doi:10.1186/s12936-016-1089-9
21. Liew, J. W. K., Mahpot, R. B., Dzul, S., Abdul Razak, H. A. B., Ahmad Shah Azizi, N. A. B., Kamarudin, M. B., . . . Lau, Y. L. (2018). Importance of Proactive Malaria Case Surveillance and Management in Malaysia. *Am J Trop Med Hyg, 98*(6), 1709-1713. doi:10.4269/ajtmh.17-1010
22. Linn, A. M., Ndiaye, Y., Hennessee, I., Gaye, S., Linn, P., Nordstrom, K., & McLaughlin, M. (2015). Reduction in symptomatic malaria prevalence through proactive community treatment in rural Senegal. *Trop Med Int Health, 20*(11), 1438-1446. doi:10.1111/tmi.12564
23. Lu, G., Liu, Y., Wang, J., Li, X., Liu, X., Beiersmann, C., . . . Müller, O. (2018). Malaria training for community health workers in the setting of elimination: a qualitative study from China. *Malar J, 17*(1), 95. doi:10.1186/s12936-018-2229-1
24. Ma, S., Lawpoolsri, S., Soonthornworasiri, N., Khamsiriwatchara, A., Jandee, K., Taweeseneepitch, K., . . . Kaewkungwal, J. (2016). Effectiveness of Implementation of Electronic Malaria Information System as the National Malaria Surveillance System in Thailand. *JMIR Public Health Surveill, 2*(1), e20. doi:10.2196/publichealth.5347

25. O'Sullivan, M., Kenilorea, G., Yamaguchi, Y., Bobogare, A., Losi, L., Atkinson, J. A., . . . Wijesinghe, R. (2011). Malaria elimination in Isabel Province, Solomon Islands: establishing a surveillance-response system to prevent introduction and reintroduction of malaria. *Malar J, 10*, 235. doi:10.1186/1475-2875-10-235
26. Pava, Z., Handayuni, I., Trianty, L., Utami, R. A. S., Tirta, Y. K., Puspitasari, A. M., . . . Auburn, S. (2017). Passively versus Actively Detected Malaria: Similar Genetic Diversity but Different Complexity of Infection. *Am J Trop Med Hyg, 97*(6), 1788-1796. doi:10.4269/ajtmh.17-0364
27. Pongvongsa, T., Nonaka, D., Iwagami, M., Nakatsu, M., Phongmany, P., Nishimoto, F., . . . Kano, S. (2016). Household clustering of asymptomatic malaria infections in Xepon district, Savannakhet province, Lao PDR. *Malar J, 15*(1), 508. doi:10.1186/s12936-016-1552-7
28. Rajvanshi, H., Bharti, P. K., Nisar, S., Jain, Y., Jayswar, H., Mishra, A. K., . . . Lal, A. A. (2020). Study design and operational framework for a community-based Malaria Elimination Demonstration Project (MEDP) in 1233 villages of district Mandla, Madhya Pradesh. *Malar J, 19*(1), 410. doi:10.1186/s12936-020-03458-4
29. Ray, A. P. W. H. O. (1964). *The discipline and dynamics of active case detection procedure under surveillance operations in a malaria eradication programme*. Retrieved from World Health Organization: https://apps.who.int/iris/handle/10665/65196
30. Rossi, G., Vernaeve, L., Van den Bergh, R., Nguon, C., Debackere, M., Abello Peiri, C., . . . Kindermans, J. M. (2018). Closing in on the Reservoir: Proactive Case Detection in High-Risk Groups as a Strategy to Detect Plasmodium falciparum Asymptomatic Carriers in Cambodia. *Clin Infect Dis, 66*(10), 1610-1617. doi:10.1093/cid/cix1064
31. Schellenberg, D. M., Aponte, J. J., Kahigwa, E. A., Mshinda, H., Tanner, M., Menendez, C., & Alonso, P. L. (2003). The incidence of clinical malaria detected by active case detection in children in Ifakara, southern Tanzania. *Trans R Soc Trop Med Hyg, 97*(6), 647-654. doi:10.1016/s0035-9203(03)80096-2
32. Singh, N., Bharti, P. K., & Kumre, N. S. (2016). Active v. passive surveillance for malaria in remote tribal belt of Central India: Implications for malaria elimination. *Pathog Glob Health, 110*(4-5), 178-184. doi:10.1080/20477724.2016.1223920

33. Taffon, P., Rossi, G., Kindermans, J. M., Van den Bergh, R., Nguon, C., Debackere, M., . . . Venables, E. (2018). 'I could not join because I had to work for pay.': A qualitative evaluation of falciparum malaria pro-active case detection in three rural Cambodian villages. *PLoS One, 13*(4), e0195809. doi:10.1371/journal.pone.0195809
34. Tang, L. H., Qian, H. L., Cui, G., Shang, L. Y., Tang, L. Y., Jiang, M. G., . . . Jin, J. Y. (1997). Study of simplified measures for malaria surveillance in the late consolidation phase in China. *Southeast Asian J Trop Med Public Health, 28*(1), 4-11.
35. Tiono, A. B., Kangoye, D. T., Rehman, A. M., Kargougou, D. G., Kaboré, Y., Diarra, A., . . . Sirima, S. B. (2014). Malaria incidence in children in South-West Burkina Faso: comparison of active and passive case detection methods. *PLoS One, 9*(1), e86936. doi:10.1371/journal.pone.0086936
36. Tseroni, M., Georgitsou, M., Baka, A., Pinaka, O., Pervanidou, D., Tsironi, M., . . . Hadjichristodoulou, C. (2020). The Importance of an Active Case Detection (ACD) Programme for Malaria among Migrants from Malaria Endemic Countries: The Greek Experience in a Receptive and Vulnerable Area. *Int J Environ Res Public Health, 17*(11). doi:10.3390/ijerph17114080
37. Vitor-Silva, S., Siqueira, A. M., de Souza Sampaio, V., Guinovart, C., Reyes-Lecca, R. C., de Melo, G. C., . . . Lacerda, M. V. (2016). Declining malaria transmission in rural Amazon: changing epidemiology and challenges to achieve elimination. *Malar J, 15*(1), 266. doi:10.1186/s12936-016-1326-2
38. Wang, T., Zhou, S. S., Feng, J., Oo, M. M., Chen, J., Yan, C. F., . . . Tie, P. (2019). Monitoring and evaluation of intervals from onset of fever to diagnosis before "1-3-7" approach in malaria elimination: a retrospective study in Shanxi Province, China from 2013 to 2018. *Malar J, 18*(1), 235. doi:10.1186/s12936-019-2865-0
39. Webster, J., Ansariadi, Burdam, F. H., Landuwulang, C. U. R., Bruce, J., Poespoprodjo, J. R., . . . Hill, J. (2018). Evaluation of the implementation of single screening and treatment for the control of malaria in pregnancy in Eastern Indonesia: a systems effectiveness analysis. *Malar J, 17*(1), 310. doi:10.1186/s12936-018-2448-5
40. West, N., Gyeltshen, S., Dukpa, S., Khoshnood, K., Tashi, S., Durante, A., & Parikh, S. (2016). An Evaluation of the National Malaria Surveillance System of Bhutan, 2006-

2012 as It Approaches the Goal of Malaria Elimination. *Front Public Health, 4*, 167. doi:10.3389/fpubh.2016.00167

41. Whidden, C., Thwing, J., Gutman, J., Wohl, E., Leyrat, C., Kayentao, K., . . . Chandramohan, D. (2019). Proactive case detection of common childhood illnesses by community health workers: a systematic review. *BMJ Glob Health, 4*(6), e001799. doi:10.1136/bmjgh-2019-001799
42. Wickremasinghe, R., Fernando, S. D., Thillekaratne, J., Wijeyaratne, P. M., & Wickremasinghe, A. R. (2014). Importance of active case detection in a malaria elimination programme. *Malar J, 13*, 186. doi:10.1186/1475-2875-13-186
43. Ferriss EL, Dieye Y, Cissé M, et al. Evaluating programmatic reactive focal drug administration impact on malaria incidence in northern Senegal: an interrupted time series analysis. Malaria Journal. 2025;24(1).
44. Hasyim H, Heroza RI, Misnaniarti M, Idris H, Maharani FE, Dale P. Malaria Surveillance Information System using an Android Mobile Phone: A Qualitative Study in Lahat District, South Sumatra Province, Indonesia. Malaysian Journal of Medicine and Health Sciences. 2024;20(2):62-70.

Wrong outcomes

1. Hua-Yun, Z., Jun, C., Guo-Ding, Z., Wei-Ming, W., Yuan-Yuan, C., Ya-Ping, G., . . . Qi, G. (2018). [Progress of malaria elimination of Jiangsu Province, China]. *Zhongguo Xue Xi Chong Bing Fang Zhi Za Zhi, 30*(4), 364-368. doi:10.16250/j.32.1374.2018165
2. Tampi RP, Wang D, Abdulla S, et al. The 1,7-malaria reactive community-based testing and response (1,7-mRCTR) approach in Tanzania: a cost-effectiveness analysis. Infect Dis Poverty. 2024;13(1):92.
3. Suwannarong K, Cotter C, Ponlap T, et al. Assessing the acceptability and feasibility of reactive drug administration for malaria elimination in a Plasmodium vivax predominant setting: a qualitative study in two provinces in Thailand. BMC Public Health. 2023;23(1):1346.

4. Kaur J, Baharia R, Dattani M. Assessment of the quality of malaria surveillance and laboratory services for diagnosis in three districts of Gujarat state, India. Front Public Health. 2024;12:1465228.
5. Reynders M, Tweneboah A, Abbas DA, et al. Challenges in diagnosis of clinical and subclinical Plasmodium falciparum infections in Ghana and feasibility of reactive interventions to shrink the subclinical reservoir. Malar J. 2024;23(1):272.
6. Ding W, Wang D, Lu S, Zhou XN, Husain L. China's approach to malaria control and elimination: Adaptive management and policy capacity building. Global Public Health. 2025;20(1).
7. Wu DN, Zhang HX, Zhu H, et al. Epidemic characteristics of malaria cases before and after malaria elimination in Hubei Province. China Tropical Medicine. 2023;23(6):579-84.
8. Wang T, Tie P, Bai Y, Zheng Y, Wang J. Epidemiological characteristics of imported malaria after malaria elimination in Shanxi Province, 2019-2023. Chinese Journal of Epidemiology. 2024;45(10):1390-5.
9. Agbemafle EE, Kubio C, Bandoh D, et al. Evaluation of the malaria surveillance system – Adaklu District, Volta Region, Ghana, 2019. Public Health in Practice. 2023;6.
10. Xuan Thang N, Win Han O, Htike W, et al. Facilitators, barriers and acceptability of malaria reactive surveillance and response strategies in Vietnam: a mixed-methods study. BMJ Public Health. 2024;2(2):e000961.
11. Chang W, Cohen J, Wang DQ, et al. Impact of 1,7-malaria reactive community-based testing and response (1,7-mRCTR) approach on malaria prevalence in Tanzania. Infectious Diseases of Poverty. 2023;12(1).
12. Aidoo EK, Aboagye FT, Arthur G, et al. Implementation of reactive case detection strategy recommendations towards malaria elimination in the Shai Osudoku district of Ghana. DISCOVER PUBLIC HEALTH. 2024;21(1).
13. Rotejanaprasert C, Malaphone V, Mayxay M, et al. Malaria epidemiology, surveillance and response for elimination in Lao PDR. Infect Dis Poverty. 2024;13(1):35.
14. Schaffner SF, Badiane A, Khorgade A, et al. Malaria surveillance reveals parasite relatedness, signatures of selection, and correlates of transmission across Senegal. Nat Commun. 2023;14(1):7268.

15. He Z, Kou Y, Wang D, et al. Neglected Time Intervals Before "1-3-7" Approach About Imported Malaria Cases - China, 2014-2021. China CDC Wkly. 2024;6(17):363-7.
16. Abossie A, Getachew H, Demissew A, et al. Profiling vivax malaria incidence, residual transmission, and risk factors using reactive case detection in low transmission settings of Ethiopia. Malar J. 2024;23(1):362.
17. Qi GAO. Risk and countermeasures of re-establishment after malaria elimination in China. China Tropical Medicine. 2024(12):1-.

Wrong settings

1. Coleman, M., Coleman, M., Mabuza, A. M., Kok, G., Coetzee, M., & Durrheim, D. N. (2009). Using the SaTScan method to detect local malaria clusters for guiding malaria control programmes. *Malar J, 8*, 68. doi:10.1186/1475-2875-8-68
2. Meankaew, P., Kaewkungwal, J., Khamsiriwatchara, A., Khunthong, P., Singhasivanon, P., & Satimai, W. (2010). Application of mobile-technology for disease and treatment monitoring of malaria in the "Better Border Healthcare Programme". *Malar J, 9*, 237. doi:10.1186/1475-2875-9-237
3. Wang, J. Z., Yin, S. Q., Li, S. G., Li, X. S., Cai, W. B., & Feng, J. (2016). Evaluation of Measures and Achievements of Malaria Control in Tengchong City, Yunnan Province during 2010-2015. *Zhongguo Ji Sheng Chong Xue Yu Ji Sheng Chong Bing Za Zhi, 34*(5), 435-438.
4. Wang, W. M., Cao, J., Zhou, H. Y., Liu, Y. B., Zhu, G. D., Cao, Y. Y., . . . Gao, Q. (2014). [Surveillance of malaria in provincial surveillance sites in Jiangsu Province, 2013]. *Zhongguo Xue Xi Chong Bing Fang Zhi Za Zhi, 26*(4), 382-386.
5. Seneviratne S, Fernando D, Wickremasinghe R, et al. An epidemiological analysis of severe imported malaria infections in Sri Lanka, after malaria elimination. Malaria Journal. 2024;23(1).

Wrong study designs

1. Gerardin, J., Bever, C. A., Bridenbecker, D., Hamainza, B., Silumbe, K., Miller, J. M., . . . Wenger, E. A. (2017). Effectiveness of reactive case detection for malaria elimination in

three archetypical transmission settings: a modelling study. *Malar J, 16*(1), 248. doi:10.1186/s12936-017-1903-z

2. Reiker, T., Chitnis, N., & Smith, T. (2019). Modelling reactive case detection strategies for interrupting transmission of Plasmodium falciparum malaria. *Malar J, 18*(1), 259. doi:10.1186/s12936-019-2893-9
3. Rosas-Aguirre, A., Erhart, A., Llanos-Cuentas, A., Branch, O., Berkvens, D., Abatih, E., . . . Speybroeck, N. (2015). Modelling the potential of focal screening and treatment as elimination strategy for Plasmodium falciparum malaria in the Peruvian Amazon Region. *Parasit Vectors, 8*, 261. doi:10.1186/s13071-015-0868-4
4. Chiziba C, Chiwaula J, Hamainza B, Silal S. Advancing malaria reactive case detection in a Zambia-like setting: A modeling study. PLOS Glob Public Health. 2025;5(2):e0004288.
5. Newby G, Cotter C, Roh ME, et al. Correction to: Testing and treatment for malaria elimination: a systematic review (Malaria Journal, (2023), 22, 1, (254), 10.1186/s12936-023-04670-8). Malaria Journal. 2024;23(1).
6. Byrne I, Nelli L, Ureña K, et al. Incorporating Community Case Management in Risk-Based Surveillance for Malaria Elimination in the Dominican Republic. Am J Trop Med Hyg. 2025;112(4):775-83.
7. Newby G, Cotter C, Roh ME, et al. Testing and treatment for malaria elimination: a systematic review. Malar J. 2023;22(1):254
