# Supplementary material for: Implementation and fidelity of reactive surveillance and response strategies for malaria elimination: a systematic review and meta-analysis
Source: BMJ Public Health. 2025 Nov 13;3(2):e001180. doi: 10.1136/bmjph-2024-001180 (PMC12625913; doi:10.1136/bmjph-2024-001180)
Supplement: online supplemental file 3 [file bmjph-3-2-s003.pdf]

Supplementary Material Three - Data extraction proforma

#	Author	Year of publication	Country	Design	Case notification	Case investigation	Reactive case detection	Focus investigation	Focus response	Other reactive surveillance and response activies	Overall reactive surveillance and response strategy
1	Cara Smith Gueye et al	2013	14 Asia Pacific countries	Cross-sectional study and desk review	No	Yes	Yes	No	Yes	No	Yes
2	Kinley Wangdi et al	2016	Bhutan	Quasi-experimental study with control	No	No	Yes	No	No	No	No
3	Gabriele Rossi et al	2017	Cambodia	Cohort study	No	No	Yes	No	No	No	Yes
4	Soy Ty Kheang et al	2020	Cambodia	Cross-sectional study	Yes	Yes	Yes	Yes	Yes	Yes	Yes
5	John Hustedt et al	2016	Cambodia	Cross-sectional study	No	Yes	Yes	No	No	No	No
6	Dysoley Lek et al	2020	Cambodia	Workshop report	No	Yes	Yes	Yes	No	Yes	Yes
7	Duoquan Wang et al	2017	China	Cross-sectional and qualitative study	Yes	Yes	Yes	No	No	No	Yes
8	Guangyu Lu et al	2016	China	Qualitative study	Yes	Yes	Yes	Yes	Yes	No	Yes
9	Shui-Sen Zhou et al	2015	China	Cross-sectional study	Yes	Yes	Yes	Yes	Yes	No	No
10	Wei Chun et al	2020	China	Cross-sectional study	Yes	Yes	No	Yes	Yes	No	Yes
11	Jun Feng et al	2018	China	Case-control study	No	No	Yes	Yes	Yes	No	Yes
12	Xiao Hui-hui et al	2015	China	Quasi-experimental study without control	No	No	Yes	No	No	No	No
13	Jun Feng et al	2016	China	Case-control study	Yes	Yes	Yes	Yes	Yes	No	No
14	Wang Wei-ming et al	2014	China	Cross-sectional study	Yes	Yes	No	Yes	Yes	No	No
15	Wang Wei-ming et al	2015	China	Secondary data analysis	Yes	Yes	No	Yes	Yes	No	Yes
16	Haung Xiao-mei et al	2018	China	Secondary data analysis	Yes	Yes	No	Yes	Yes	No	Yes
17	Christ Cotter et al	2017	China, Indonesia and	Quasi-expenimental study without control	Yes	Yes	Yes	No	No	Yes	Yes

18	B. Shantharam Baliga et al	2019	India	Quasi-experimental study without control	Yes	No	No	Yes	Yes	Yes	No
19	Brittany W. Zelman et al	2018	Indonesia	Economic analysis	No	No	Yes	No	No	No	No
20	Herdiana Herdiana et al	2016	Indonesia	Cross-sectional study	Yes	Yes	Yes	No	No	No	No
21	PMI Impact Malaria Project	2022	Lao PDR	Qualitative study	Yes	Yes	No	Yes	Yes	No	Yes
22	Aye Mon Mon Kyaw et al	2018	Myanmar	Cohort study	Yes	Yes	No	No	Yes	No	No
23	Poe Poe Aung et al	2020	Myanmar	Cross-sectional and qualitative study	Yes	Yes	Yes	Yes	Yes	Yes	Yes
24	Daniel M. Parker et al	2016	Myanmar	Randomised controlled trial	No	No	Yes	No	No	No	No
25	San Kyawt Khine et al	2019	Myanmar	Cross-sectional study	No	Yes	No	No	No	No	No
26	Elizabeth T. Rogawski et al	2012	Thailand	Cross-sectional study	No	No	Yes	No	No	No	No
27	Amnat Khamsiriwatchara et	2012	Thailand	Cross-sectional study	Yes	Yes	Yes	Yes	Yes	Yes	Yes
28	Wesley Donald et al	2016	Vanuatu	Cross-sectional study	No	No	Yes	No	No	No	No
29	Thang Duc Ngo et al	2019	Vietnam	Quasi-experimental study without control	Yes	Yes	Yes	No	No	No	Yes
30	Cavin Epie Bekolo et al	2019	Cameroon	Cohort study	No	Yes	Yes	No	No	No	No
31	Hasan Hamze et al	2016	Democratic Republic of	Cohort study	No	No	Yes	No	No	No	No
32	Endalew Zemene et al	2018	Ethiopia	Cohort study	No	No	Yes	No	No	No	No
33	Pooja Bansil et al	2018	Ethiopia	Quasi-experimental study without control	No	Yes	Yes	Yes	Yes	No	No
34	Sofonias K. Tessema et al	2020	Ethiopia	Case-control study	No	No	Yes	No	No	No	No
35	Ebenezer K. Aidoo et al	2018	Kenya	Cross-sectional study	No	Yes	Yes	Yes	No	No	No
36	Michelle S. Hsiang et al	2020	Namibia	Cluster randomised controlled trial	No	No	Yes	No	Yes	No	No
37	Munyaradzi Tambo et al	2018	Namibia	Case-control study	No	No	Yes	No	No	No	No
38	Jennifer L Smith et al	2017	Namibia	Case-control study	No	No	Yes	No	No	No	No

39	Megan Littrell et al	2013	Senegal	Cross-sectional study	Yes	Yes	Yes	No	No	No	No
40	Ruben O. Conner et al	2020	Senegal	Quasi-experimental study with control	No	Yes	Yes	Yes	Yes	No	Yes
41	Craig Davies et al	2019	South Africa	Cohort study	Yes	No	No	No	No	No	No
42	Michelle S. Hsiang et al	2020	Swaziland	Cohort study	No	Yes	Yes	No	No	No	No
43	N. Dlamini et al	2018	Swaziland	Cross-sectional study	Yes	Yes	No	No	No	No	No
44	Hugh J. W. Sturrock et al	2013	Swaziland	Cohort study	No	Yes	Yes	No	No	No	Yes
45	Benjamin Grossenbacher et al	2020	Tanzania	Cross-sectional study	No	No	Yes	No	No	No	No
46	Eeshan Khandekar et al	2019	Tanzania	Cross-sectional and qualitative study	No	Yes	Yes	No	No	No	No
47	Lynne Lohfeld et al	2016	Zambia	Qualitative study	No	No	Yes	No	No	Yes	No
48	Kelly M. Searle et al	2013	Zambia	Cross-sectional study	No	No	Yes	No	No	No	No
49	Joshua Yukich et al	2017	Zambia	Cluster randomized controlled trial	No	No	Yes	No	Yes	No	No
50	Julia C Pringle et al	2019	Zambia	Quasi-experimental study without control	No	No	Yes	No	No	No	Yes
51	Fiona R. P. Bhondoekhan et al	2020	Zambia	Cross-sectional study	No	No	Yes	No	No	No	No
52	David A. Larsen et al	2017	Zambia	Case-control study	No	Yes	Yes	No	No	No	Yes
53	David A. Larsen et al	2015	Zambia	Quasi-experimental study without control	Yes	Yes	Yes	No	No	No	Yes
54	David A. Larsen et al	2017	Zambia	Cohort study	No	No	Yes	No	No	No	No
55	Jessie Pinchoff et al	2015	Zambia	Cross-sectional study	No	No	Yes	No	No	No	No
56	Daniel J. Bridges et al	2020	Zambia	Cross-sectional study	No	Yes	Yes	Yes	No	No	No
57	Nakul Chitnis et al	2019	Zambia	Cross-sectional study	No	No	Yes	No	No	No	No
58	Gillian H. Stresman et al	2010	Zambia	Case-control study	No	No	Yes	No	No	No	No
59	Kelly M. Searle et al	2016	Zambia	Cross-sectional study	No	No	Yes	No	No	No	Yes

60	Logan Stuck et al	2020	Zanzibar	Cross-sectional study	No	No	Yes	No	No	No	No
61	Tina van der Horst et al	2020	Zanzibar	Cross-sectional study	Yes	Yes	Yes	No	No	No	Yes
62	Pablo S. Fontoura et al	2016	Brazil	Randomised controlled trial	No	No	Yes	No	No	No	No
63	Karen Molina Gomez et al	2017	Colombia	Cross-sectional study	Yes	Yes	Yes	Yes	No	No	Yes
64	Martin A. C et al	2025	Zambia	Cluster randomized controlled trial	Yes	Yes	Yes	Yes	No	No	No
65	Mkali H. R. et al	2023	Zanzibar	Cross-sectional study	Yes	Yes	Yes	Yes	No	No	No
66	Zhang L. et al	2024	China	Cross-sectional study	Yes	Yes	No	Yes	No	No	Yes
67	Win Han Oo et al	2023	Vietnam	Cross-sectional study	Yes	Yes	Yes	Yes	Yes	Yes	Yes
68	Kandel S. et al	2024	Nepal	Cross-sectional study	Yes	Yes	No	No	No	No	No
69	Htike W. et al	2024	Lao PDR	Cross-sectional study	Yes	Yes	Yes	Yes	Yes	Yes	No
					29	40	57	24	21		

Case notification

ID	Auther_year	Country	T_schedule_day	T_average_day	Methods_notification	#malaria_cases	#notified	#notified_intime	%completeness	%timeliness	Remark
	Author and year of publication	country of research	Time schedule for case notification in day	Average notification time in days	Method of case notification	Number of people diagnosed for malaria	Number of people notified	Number of people notified in time	Completeness of case notification	Timeliness of case notification	Remark
921	Ngo 2019	Vietnam	2	2	By phone	128	128	128	100.00	100.00	
741	Wang 2015	China	1	1	Online reporting platform	341	341	341	100.00	100.00	
726	Xiao-Mei 2018	China	1	1	.	64	64	64	100.00	100.00	
642	Davies 2019	South Africa	1	5.65	PBR	1047	1039	5	99.24	0.48	
642	Davies 2019	South Africa	1	0.63	MalariaConnect (Web-based)	1047	704	604	67.24	57.69	
632	Dlamini 2018	Swaziland	1	.	By phone	1991	1513	1314	75.99	66.00	
403	Cotter 2017	China	1	.	.	42	42	42	100.00	100.00	
403	Cotter 2017	Indonesia	30	.	.	120	112	106	93.33	88.33	
403	Cotter 2017	Thailand	3	.	.	510	510	259	100.00	50.78	
365	Feng 2016	China	1		Web-based	858	858	858	100.00	100.00	
273	Kyaw 2018	Myanmar	1	.	PBR	959	312	.	32.53	#VALUE!	
248	Littrell 2013	Senegal	1	1	By phone or SMS	.	.	.	#VALUE!	#VALUE!	
43	VanderHorst 2020	Zanzibar	1	1.8	By phone	1305	1126	.	86.28	#VALUE!	
34	Wang 2017	China	1	1	Web-based	260	260	260	100.00	100.00	
25	Wei 2020	China	1	1	Web-based	2283	2283	2283	100.00	100.00	
924	Martin 2025	Zambia	1	1	TextIt (SMS)	41	34	25	82.93	61	
923	Mkali 2023	Zanzibar	1		MCN (SMS)	48899		22,152		45.30	Zanzibar Malaria Case Notification platform

922	Zhang 2024	China	1		4132		4132		100
925	Oo 2023	Vietnam	2	paper-based	12498	12463	7978	99.72	63.83
925	Oo 2023	Vietnam	2	Web-based	467	467	391	100.00	83.73
926	Kandel 2024	Nepal	1	SMS	2547		2161		84.84
927	Htike 2024	Laos	1	reporting or paper-based or phone	796		249		31.28

Case investigation

ID	Auther_year	Country	schedule_day	_average_day	Methods_CI	#malaria_cases	#notified	#CI_intime	%completeness	%timeliness
	and year of publication	ntry of research								
			igation in day	n time in days	of case investigation	sed for malaria	le investigated	tigated in time	e investigation	e investigation
741	Wang 2015	China	3	2	.	341	341	341	100.00	100.00
726	Xiao-Mei 2018	China	3	.	.	18076	18076	18076	100.00	100.00
704	Bansil 2018	Ethiopia	.	.	rview questionnaire	407	220	.	54.05	#VALUE!
656	Conner 2020	Senegal	.	1.3	t and data collection	13	13	.	100.00	#VALUE!
632	Dlamini 2018	Swaziland	2	6	t and data collection	1991	1353	270	67.96	13.56
525	Khine 2019	Myanmar	3		Home visit and data collection	175	157	.	89.71	#VALUE!
403	Cotter 2017	China	3	.	.	42	42	42	100.00	100.00
403	Cotter 2017	Indonesia	30	.	.	111	87	79	78.38	71.17
403	Cotter 2017	Thailand	3	.	.	752	465	394	61.84	52.39
365	Feng 2016	China	3	.	.	.	.	.	96.30	96.30
317	Hsiang 2020	Eswatini	2	.	t and data collection	1394	1163	.	83.43	#VALUE!
273	Kyaw 2018	Myanmar	3	.	t and data collection	959	312	298	32.53	31.07
269	Larsen 2017	Zambia	.	.	RDT testing	2469	854	.	34.59	#VALUE!
248	Littrell 2013	Senegal	3	3	t and data collection	110	110	110	100.00	100.00
61	Sturrock 2013	Swaziland	ASAP	.	t and data collection	1002	675	.	67.37	#VALUE!
43	nderHorst 2020	Zanzibar	3	.	t and data collection	1275	790	450	61.96	35.29
34	Wang 2017	China	3	.	t and data collection	260	260	212	100.00	81.54
31	Wang 2014	China	3	.	t and data collection	94	94	82	100.00	87.23
25	Wei 2020	China	3	.	t and data collection	2283	2283	2283	100.00	100.00
3	Zhou 2015	China	3	.	t and data collection	576	576	539	100.00	93.58
924	Martin 2025	Zambia	3	3.9	t and data collection	41	29	18	70.73	44.00
923	Mkali 2023	Zanzibar	3		Home visit and data collection	48899	41,886	32097	85.66	76.63
922	Zhang 2024	China	3			4132		3970		96.10
925	Oo 2023	Vietnam	3		Home visit and data collection	12498	11766		94.14	
925	Oo 2023	Vietnam	2		Home visit and data collection	467	453	371	97.00	79.44
926	Kandel 2024	Nepal	3			2893		2611		90.25
927	Htike 2024	Laos	3		Home visit and data collection	796		517		64.95

Reactive case detection (RACD)

ID	Auther_year	Country	RACD_Period_Month	Diagnosis	T_schedule_day	T_average_day	#tested_in time	#RACD_event_in time	#RACD_event_total	%timeliness	%completeness	Radius_M	Index_eligible	#Index_intervened	#eligible_RACD_total	#tested_total	#secondary_cases_total	%RACD_overall	#tested_index_hh	#secondary_cases_index_hh	%RACD_index_hh	#tested_neighbour_hh	#secondary_cases_neighbour_hh	%RACD_neighbour_hh	#tested_control_hh	#secondary_cases_control_hh	%RACD_control_hh
	Author and year of publication	country of research	RACD implementation period in months	Diagnostic test used for malaria	Time schedule for reactive case detection in days	Average RACD time for reactive case detection in days	# of people tested in time	# of RACD events performed in time	Total RACD events occurred	Timeliness of reactive case detection in percentage	Completeness of reactive case detection	Radius of screening	Number of index cases identified in PCD/ health facility	Number of index cases followed up and taken action with RACD	Total number of people eligible for RACD	Total number of people tested for malaria	Total number of malaria positive (secondary) cases	Overall positivity rate of RACD	Number of people tested in index households	Number of malaria positive (secondary) cases in the index household	Positivity rate of RACD in index households	Number of people tested in neighbour households	Number of malaria positive (secondary) cases in the neighbour household	Positivity rate of RACD in neighbour households	Number of people tested in control households	Number of malaria positive (secondary) cases in the control household	Positivity rate of RACD in control households
921	Ngo 2019	Vietnam	24	RDT	3	.	.	.	.	.	10.66	Whole village	.	128	17,563	1872	0	0	.	.	.	.	.	.	.	.	#VALUE!
704	Bansil 2018	Ethiopia	5	RDT	.	.	.	.	.	#VALUE!	84.10	100	.	407	3856	3243	127	3.92	772	37	4.79	2471	90	3.64	.	.	#VALUE!
656	onner 2020	Senegal	4	RDT	.	1.3	.	.	.	#VALUE!	96.59	100	.	13	205	198	7	3.54	198	7	3.54	18,992	291	1.53	.	.	#VALUE!
497	Aidoo 2018	Kenya	12	Microscopy	7	.	.	.	.	#VALUE!	56.25	100	.	50	1280	720	72	10	108	9	8.33	612	63	10.29	510	24	4.71
497	Aidoo 2018	Kenya	12	PCR	7	.	.	.	.	#VALUE!	65.47	100	.	50	1280	838	213	25.42	360	93	25.83	478	120	25.10	263.00	34.00	12.93
468	Baliga 2019	India	60	RDT + Microscopy	.	.	.	.	.	#VALUE!	#VALUE!	Whole city	.	.	.	549290	40083	7.30	.	.	#VALUE!	.	.	#VALUE!	.	.	#VALUE!
465	sema 2020	Ethiopia	3	RDT	2	.	.	.	.	#VALUE!	97.80	6 nearest neighboring household	.	18	499	488	64	13.11	.	.	#VALUE!	.	.	#VALUE!	444.00	26.00	5.86
465	sema 2020	Ethiopia	3	PCR	2	.	.	.	.	#VALUE!	92.18	6 nearest neighboring household	.	18	499	460	111	24.13	.	.	#VALUE!	.	.	#VALUE!	429.00	89.00	20.75
443	ekolo 2019	Cameroon	3	RDT	.	.	.	.	.	#VALUE!	#VALUE!	Index household	.	36	.	290	249	85.86	290	249	85.86	.	.	#VALUE!	.	.	#VALUE!
436	ekhan 2020	Zambia	31	RDT or PCR	.	.	.	.	.	#VALUE!	#VALUE!	250	.	158	.	4170	531	12.73	.	.	#VALUE!	.	.	#VALUE!	.	.	#VALUE!
432	idges 2020	Zambia	60	RDT + PCR	7	.	.	.	.	#VALUE!	4.91	ouseholds	8723	428	.	11954	206	1.72	.	.	#VALUE!	.	.	#VALUE!	.	.	#VALUE!
403	Cotter 2017	a, Thailand	3	RDT + Microscopy + PCR	7	.	295	295	348	84.77	76.01	100	496	377	.	19436	29	0.15	.	.	#VALUE!	.	.	#VALUE!	.	.	#VALUE!
383	onald 2016	Vanuatu	1	RDT	5	.	.	.	.	#VALUE!	#VALUE!	500	.	2	.	126	0	0.00	.	.	#VALUE!	.	.	#VALUE!	.	.	#VALUE!
383	onald 2016	Vanuatu	1	PCR	5	.	.	.	.	#VALUE!	#VALUE!	500	.	2	.	126	1	0.79	.	.	#VALUE!	.	.	#VALUE!	.	.	#VALUE!
384	onald 2016	Vanuatu	1	RDT	5	.	.	.	.	#VALUE!	#VALUE!	500	.	2	.	47	0	0.00	.	.	#VALUE!	.	.	#VALUE!	.	.	#VALUE!

385	onald 2016	Vanuatu	1	PCR	5	.	.	.	.	#VALUE!	#VALUE!	500	.	2	.	47	0	0.00	.	.	#VALUE!	.	.	#VALUE!	.	.	#VALUE!
365	Feng 2016	China	120	RDT + Microscopy + PCR	7	8.5	858	.	.	100.00	100.00	.	.	858	858	858	0	0.00	.	.	#VALUE!	.	.	#VALUE!	.	.	#VALUE!
364	Feng 2018	China	72		7	.	81	.	.	96.43	83.17	.	.	28	101	84	4	4.76	.	.	#VALUE!	.	.	#VALUE!	.	.	#VALUE!
359	Fontoura 2	Brazil	7	Microscopy	.	.	.	.	.	.	.	3000	.	41	1923	3280	105	3.20	577	35	6.07	2703	70	2.59	2586	3	0.12
359	Fontoura 2	Brazil	7	PCR	.	.	.	.	.	.	.	3000	.	41	1923	3240	212	6.54	575	61	10.61	2665	151	5.67	2567	81	3.16
340	Grossenbac	Tanzania	14	PCR	.	.	.	.	.	#VALUE!	100.00	200	.	156	4590	4590	78	1.70	664	.	#VALUE!	1955	.	#VALUE!	1971	.	#VALUE!
340	Grossenbac	Tanzania	14	RDT	.	.	.	.	.	#VALUE!	100.00	200	.	156	4590	4590	33	0.72	664	.	#VALUE!	1955	.	#VALUE!	1971	.	#VALUE!
334	Hamze 201	Democratic	2	RDT	.	.	.	.	.	#VALUE!	100.00	.	.	19	68	68	5	7.35	68	5	7.35	.	.	#VALUE!	294	24	8.16
327	Herdiana 2	Indonesia	18	Microscopy	7	.	1440	.	.	96.32	91.27	500	.	38	1638	1495	3	0.20	.	.	#VALUE!	.	.	#VALUE!	.	.	#VALUE!
327	Herdiana 2	Indonesia	18	PCR	7	.	1440	.	.	96.32	91.27	500	.	38	1638	1495	6	0.40	.	.	#VALUE!	.	.	#VALUE!	.	.	#VALUE!
317	Hsiang 202	Eswatini	31	RDT	35	.	.	.	.	#VALUE!	91.67	500	.	1163	10890	9983	65	0.65	.	.	#VALUE!	.	.	#VALUE!	.	.	#VALUE!
317	Hsiang 202	Eswatini	31	LAMP	35	.	.	.	.	#VALUE!	95.92	500	.	1163	10890	10446	180	1.72	.	.	#VALUE!	.	.	#VALUE!	.	.	#VALUE!
316	Hsiang 202	Namibia	12	RDT	35	.	.	.	.	#VALUE!	88.77	500	.	708	5296	4701	114	2.43	.	.	#VALUE!	.	.	#VALUE!	.	.	#VALUE!
312	Hustedt 20	Cambodia	11	RDT	3	.	.	.	.	#VALUE!	#VALUE!	ouseholds	.	270	.	1898	9	0.47	1266	4	0.32	632	5	0.79	183	0	0.00
312	Hustedt 20	Cambodia	11	PCR	3	.	.	.	.	#VALUE!	#VALUE!	ouseholds	.	270	.	1596	17	1.07	1047	13	1.24	549	4	0.73	237	25	10.55
284	Kheang 202	Cambodia	18	RDT	7	.	.	.	.	#VALUE!	100.00	o-travellers	.	408	1377	1377	14	1.02	900	0	0.00	477	14	2.94	.	.	#VALUE!
270	Larsen 201	Zambia	12	RDT	7			.	.	0.00	#VALUE!	140	.	144	.	3,955	66	1.67			#DIV/0!			#DIV/0!			#DIV/0!
269	Larsen 201	Zambia	24	RDT	.	.	.	.	.	#VALUE!	34.59	140	2469	854	.	14409	1200	8.33	.	.	#VALUE!	.	.	#VALUE!	.	.	#VALUE!
260	Lek 2020	Cambodia	24	RDT	14	.	.	.	.	#VALUE!	#VALUE!	.	.	1161	.	5644	52	0.92	.	.	#VALUE!	.	.	#VALUE!	.	.	#VALUE!

260	Lek 2020	Cambodia	24	PCR	14	.	.	.	.	#VALUE!	#VALUE!	.	.	1161	.	5644	91	1.61	.	.	#VALUE!	.	.	#VALUE!	.	.	#VALUE!
248	Littrell 201	Senegal	3	RDT	.	.	.	.	.	#VALUE!	#VALUE!	0 - 500 M)	.	110	.	5520	23	0.42	.	.	#VALUE!	.	.	#VALUE!	.	.	#VALUE!
211	MolinaGÃ³ı	Colombia	1	PCR	.	.	.	.	.	#VALUE!	#VALUE!	households	.	16	.	289	41	14.19	.	.	#VALUE!	.	.	#VALUE!	.	.	#VALUE!
211	MolinaGÃ³ı	Colombia	1	Microscopy	.	.	.	.	.	#VALUE!	#VALUE!	households	.	16	.	289	11	3.81	.	.	#VALUE!	.	.	#VALUE!	.	.	#VALUE!
161	Pinchoff 20	Zambia	13	RDT	.	.	.	.	.	#VALUE!	13.92	household	3061	426	.	1621	735	45.34	1621	735	45.34	.	.	#VALUE!	.	.	#VALUE!
151	Pringle 201	Zambia	16	RDT	7	.	.	.	.	#VALUE!	#VALUE!	250	.	27	.	106	58	54.72	.	.	#VALUE!	.	.	#VALUE!	.	.	#VALUE!
135	Rogawski 2	Thailand	1	Microscopy	14	14	187	.	.	100.00	100.00	and MMPs	.	1	187	187	1	0.53	.	.	#VALUE!	.	.	#VALUE!	.	.	#VALUE!
135	Rogawski 2	Thailand	1	PCR	14	14	187	.	.	100.00	100.00	and MMPs	.	1	187	187	4	2.14	126	2	1.59	61	2	3.28	.	.	#VALUE!
128	Rossi 2018	Cambodia	18	RDT	.	.	.	.	.	#VALUE!	#VALUE!	ed persons	.	194	.	785	7	0.89	623	1	0.16	162	6	3.70	.	.	#VALUE!
128	Rossi 2018	Cambodia	18	PCR	.	.	.	.	.	#VALUE!	#VALUE!	ed persons	.	194	.	785	31	3.95	623	20	3.21	162	11	6.79	.	.	#VALUE!
101	Searle 201	Zambia	12	RDT	7	3	.	.	.	#VALUE!	30.66	140	411	126	.	428	50	11.68	171	37	21.64	257	13	5.06	.	.	#VALUE!
72	Smith 2017	Namibia	18	RDT	2	14	.	.	.	#VALUE!	58.90	households	.	146	3151	1856	18	0.97	801	11	1.37	1055	7	0.66	1295	5	0.39
72	Smith 2017	Namibia	18	LAMP	2	14	.	.	.	#VALUE!	58.90	households	.	146	3151	1856	42	2.26	801	27	3.37	1055	15	1.42	1295	5	0.39
64	Stresman 2	Zambia	3	RDT	14	.	.	.	.	#VALUE!	#VALUE!	households	.	.	.	#VALUE!	#VALUE!	#VALUE!	185	5	2.70	.	.	#VALUE!	.	.	#VALUE!
64	Stresman 2	Zambia	3	PCR	14	.	.	.	.	#VALUE!	#VALUE!	households	.	.	.	#VALUE!	#VALUE!	#VALUE!	186	13	6.99	.	.	#VALUE!	.	.	#VALUE!
63	Stuck 2020	Zanzibar	12	RDT	.	5	.	.	.	#VALUE!	71.47	households	.	406	17,458	12,478	104	0.83	1917	62	3.23	10561	42	0.40	.	.	#VALUE!
63	Stuck 2020	Zanzibar	12	PCR	.	5	.	.	.	#VALUE!	35.98	households	.	406	17,458	6,281	148	2.36	953	80	8.39	5328	68	1.28	.	.	#VALUE!
61	Sturrock 2C	Swaziland	31	RDT	.	7	.	.	.	#VALUE!	24.95	1000	1002	250	.	3671	74	2.02	1702	56	3.29	1529	12	0.78	.	.	#VALUE!
56	Tambo 201	Namibia	20	RDT	.	.	.	.	.	#VALUE!	83.85	households	.	.	3151	2642	23	0.87	.	.	#VALUE!	.	.	#VALUE!	.	.	#VALUE!

56	Tambo 201	Namibia	20	LAMP	.	.	.	.	.	#VALUE!	83.78	households	.	.	3151	2640	47	1.78	.	.	#VALUE!	.	.	#VALUE!	.	.	#VALUE!
56	Tambo 201	Namibia	20	PCR	.	.	.	.	.	#VALUE!	10.00	households	.	.	3151	315	43	13.65	.	.	#VALUE!	.	.	#VALUE!	.	.	#VALUE!
43	VanderHor	Zanzibar	24	RDT	3	3	450	450	1275	35.29	86.28	households	1305	1126	.	#VALUE!	#VALUE!	#VALUE!	.	.	#VALUE!	.	.	#VALUE!	.	.	#VALUE!
34	Wang 2017	China	36	RDT + Microscop y	7	7	164	.	.	4.48	11.66	hole village	260	182	31408	3662	10	0.27	.	.	#VALUE!	.	.	#VALUE!	.	.	#VALUE!
29	Wangdi 20:	Bhutan	8	RDT + Microscop y	.	.	.	.	.	#VALUE!	10.16	1000	.	6	7165	728	#VALUE!	#VALUE!	.	.	#VALUE!	.	.	#VALUE!	.	.	#VALUE!
17	Xiao 2015	China	.	PCR	7	.	.	.	.	#VALUE!	#VALUE!	300	.	.	.	278	6	2.16	64	2	3.13	214	4	1.87	.	.	#VALUE!
17	Xiao 2015	China	.	Microscop y	7	.	.	.	.	#VALUE!	#VALUE!	300	.	.	.	278	3	1.08	.	.	#VALUE!	.	.	#VALUE!	.	.	#VALUE!
9	Zelman 201	Indonesia	20	Microscop y	7	.	.	.	.	#VALUE!	91.38	0 - 500 M)	.	36	1636	1495	3	0.20	.	.	#VALUE!	.	.	#VALUE!	.	.	#VALUE!
9	Zelman 201	Indonesia	20	LAMP	7	.	.	.	.	#VALUE!	91.38	0 - 500 M)	.	36	1636	1495	7	0.47	.	.	#VALUE!	.	.	#VALUE!	.	.	#VALUE!
9	Zelman 201	Indonesia	20	PCR	7	.	.	.	.	#VALUE!	91.38	0 - 500 M)	.	36	1636	1495	6	0.40	.	.	#VALUE!	.	.	#VALUE!	.	.	#VALUE!
8	Zemene 20	Ethiopia	6	Microscop y	7	.	.	.	.	#VALUE!	#VALUE!	200	.	39	.	726	29	3.99	.	.	#VALUE!	.	.	#VALUE!	.	.	#VALUE!
8	Zemene 20	Ethiopia	6	PCR	7	.	.	.	.	#VALUE!	#VALUE!	200	.	39	.	603	54	8.96	120	21	17.50	483	33	6.83	.	.	#VALUE!
3	Zhou 2015	China	18	PCR	7	.	53	.	.	#VALUE!	96.36	.	55	53	.	.	.	.	.	.	.	.	.	.	.	.	.
924	'artin 2025	Zambia		RDT	7	6.8	8			20	31.70732	140	41	13	1595				312								
923	Mkali 2023	Zanzibar	120	RDT	7		32,257			65.96658	79.68466	households	48899	38,965					111811	10602	9.5						
922	Zhang 2024	China			7		3950			95.6			4132														
925	Oo 2023	Vietnam		RDT + Microscop y + PCR	7						88	households															

Legend

Method of diagnosis	Abbreviation
Rapid Diagnostic Test	RDT
Malaria microscopy	Microscopy
Polymerase Chain Reaction	PCR
RDT and microscopy	RDT + Microscopy
Microscopy and PCR	Microscopy + PCR
RDT and microscopy and PCR	RDT + Microscopy + PCR
RDT and PCR	RDT + PCR
LAMP	LAMP
Others	Others
