# Supplementary material for: Implementation and fidelity of reactive surveillance and response strategies for malaria elimination: a systematic review and meta-analysis
Source: BMJ Public Health. 2025 Nov 13;3(2):e001180. doi: 10.1136/bmjph-2024-001180 (PMC12625913; doi:10.1136/bmjph-2024-001180)
Supplement: online supplemental file 4 [file bmjph-3-2-s004.pdf]

Supplementary Material 4: Formulae for calculation of outcomes

Estimates for completeness and timeliness of case notification and case investigation were shown as proportions of total people tested for malaria. Detail formulae are shown below.

Completeness of case notification = (number of people notified)/ (number of people diagnosed for malaria)

Timeliness of case notification = (number of people notified in time*)/ (number of people diagnosed for malaria)

Completeness of case investigation = (number of people investigated)/ (number of people diagnosed for malaria)

Timeliness of case investigation = (number of people investigated in time*)/ (number of people diagnosed for malaria)

With regards to completeness and timeliness of RACD, reviewed study articles had differing calculations. It is noted that in estimating the timeliness indicators in each study, time frame is various as defined by the policy of national malaria programme. Hence, corresponding values for the numerator and denominator used to estimate proportions were extracted manually based on available data as in the following formulae.

Completeness of RACD = (number of people reactively tested for malaria triggered by index cases)/ (number of people eligible for RACD) or

Completeness of RACD = (number of index cases followed up and taken action with RACD)/ (number of index cases identified via passive case detection)

Timeliness of RACD = (number of people reactively tested in time*)/ (number of people tested for malaria) or

Timeliness of RACD = (number of RACD events performed in time*)/ (number of RACD events occurred)

*As per the policy of national malaria programmes
