# Supplementary material for: Implementation and fidelity of reactive surveillance and response strategies for malaria elimination: a systematic review and meta-analysis
Source: BMJ Public Health. 2025 Nov 13;3(2):e001180. doi: 10.1136/bmjph-2024-001180 (PMC12625913; doi:10.1136/bmjph-2024-001180)
Supplement: online supplemental file 5 [file bmjph-3-2-s005.pdf]

Supplementary material 5: Supplementary tables and figures

Characteristics of the included studies

Among the included studies (n = 69), 29 reported case notification (n = 21 in the Asia Pacific, n = 7 in Africa and n = 1 South America Regions), 40 studies reported case investigation (n = 24 in the Asia Pacific, n = 15 in Africa and n = 1 in South America Regions), 57 studies reported Reactive Case Detection (RACD) (n = 23 in the Asia Pacific, n = 32 in Africa and n = 2 in South America), 24 studies reported other foci investigation (n = 17 in the Asia Pacific, 6 in Africa and n = 1 South America Regions), and 21 studies reported focus responses (n=17 in the Asia Pacific, 4 in Africa Regions) **(Supplementary Table 1)**.

Supplementary Table 1: Characteristics of the 69 included studies that report reactive surveillance and response strategies

Author	Year*	Country	Study design	CN	CI	RACD	FI	FR	Strategy
			**Asia-Pacific Region**						
Cara Smith Gueye et al [1]	2013	14 Asia Pacific countries	Cross-sectional study and desk review	No	Yes	Yes	No	Yes	1-3-7
Kinley Wangdi et al [2]	2016	Bhutan	Quasi-experimental study with control	No	No	Yes	No	No	RACD
Gabriele Rossi et al [3]	2018	Cambodia	Cohort study	No	No	Yes	No	No	RACD
Soy Ty Kheang et al [4]	2020	Cambodia	Cross-sectional study	Yes	Yes	Yes	Yes	Yes	1-3-7
John Hustedt et al [5]	2016	Cambodia	Cross-sectional study	No	Yes	Yes	No	No	1-3-7
Dysoley Lek et al [6]	2020	Cambodia	Workshop report	No	Yes	Yes	Yes	No	1-3-7
Duoquan Wang et al [7]	2017	China	Cross-sectional and qualitative study	Yes	Yes	Yes	No	No	1-3-7
Guangyu Lu et al [8]	2016	China	Qualitative study	Yes	Yes	Yes	Yes	Yes	1-3-7
Shui-Sen Zhou et al [9]	2015	China	Cross-sectional study	Yes	Yes	Yes	Yes	Yes	1-3-7
Wei Chun et al [10]	2020	China	Cross-sectional study	Yes	Yes	No	Yes	Yes	1-3-7
Jun Feng et al [11]	2018	China	Case-control study	No	No	Yes	Yes	Yes	1-3-7
Xiao Hui-hui et al [12]	2015	China	Quasi-experimental study without control	No	No	Yes	No	No	1-3-7
Jun Feng et al [13]	2016	China	Case-control study	Yes	Yes	Yes	Yes	Yes	1-3-7
Wang Wei-ming et al [14]	2014	China	Cross-sectional study	Yes	Yes	No	Yes	Yes	1-3-7
Wang Wei-ming et al [15]	2015	China	Secondary data analysis	Yes	Yes	No	Yes	Yes	1-3-7
Haung Xiao-mei et al [16]	2018	China	Secondary data analysis	Yes	Yes	No	Yes	Yes	1-3-7

Zhang L. et al [17]	2024	China	Cross-sectional study	Yes	Yes	No	Yes	No	1-3-7
Christ Cotter et al [18]	2017	China, Indonesia, Thailand	Quasi-experimental study without control and qualitative study	Yes	Yes	Yes	No	No	1-3-7
B. Shantharam Baliga et al [19]	2019	India	Quasi-experimental study without control	Yes	No	No	Yes	Yes	1-3-7
Brittany W. Zelman et al [20]	2018	Indonesia	Economic analysis	No	No	Yes	No	No	RACD
Herdiana et al [21]	2016	Indonesia	Cross-sectional study	Yes	Yes	Yes	No	No	1-3-7
PMI Impact Malaria Project [22]	2022	Lao PDR	Qualitative study	Yes	Yes	No	Yes	Yes	1-3-7
Htike W. et al [23]	2024	Lao PDR	Cross-sectional study	Yes	Yes	Yes	Yes	Yes	1-3-7
Aye Mon Mon Kyaw et al [24]	2018	Myanmar	Cohort study	Yes	Yes	No	No	Yes	1-3-7
Poe Poe Aung et al [25]	2020	Myanmar	Cross-sectional and qualitative study	Yes	Yes	Yes	Yes	Yes	1-3-7
Daniel M. Parker et al [26]	2016	Myanmar	Randomised controlled trial	No	No	Yes	No	No	RACD
San Kyawt Khine et al [27]	2019	Myanmar	Cross-sectional study	No	Yes	No	No	No	1-3-7
Kandel S. et al [28]	2024	Nepal	Cross-sectional study	Yes	Yes	No	No	No	1-3-7
Elizabeth T. Rogawski et al [29]	2012	Thailand	Cross-sectional study	No	No	Yes	No	No	RACD
Amnat Khamsiriwatchara et al [30]	2012	Thailand	Cross-sectional study	Yes	Yes	Yes	Yes	Yes	1-3-7
Wesley Donald et al [31]	2016	Vanuatu	Cross-sectional study	No	No	Yes	No	No	RACD
Ngo Duc Thang et al [32]	2019	Vietnam	Quasi-experimental study without control and qualitative study	Yes	Yes	Yes	No	No	1-3-7
Win Han Oo et al [33]	2023	Vietnam	Cross-sectional study	Yes	Yes	Yes	Yes	Yes	2-7 and 1-3-7
**African Region**									
Cavin Epie Bekolo et al [34]	2019	Cameroon	Cohort study	No	Yes	Yes	No	No	1-3-7
Hasan Hamze et al [35]	2016	Democratic Republic of Congo	Cohort study	No	No	Yes	No	No	RACD
Michelle S. Hsiang et al [36]	2020	Eswatini (Swaziland)	Cohort study	No	Yes	Yes	No	No	1-3-7
N. Dlamini et al [37]	2018	Eswatini (Swaziland)	Cross-sectional study	Yes	Yes	No	No	No	1-3-7
Hugh J. W. Sturrock et al [38]	2013	Eswatini (Swaziland)	Cohort study	No	Yes	Yes	No	No	1-3-7
Endalew Zemene et al [39]	2018	Ethiopia	Cohort study	No	No	Yes	No	No	RACD
Pooja Bansil et al [40]	2018	Ethiopia	Quasi-experimental study without control	No	Yes	Yes	Yes	Yes	1-3-7
Sofonias K. Tessema et al [41]	2020	Ethiopia	Case-control study	No	No	Yes	No	No	RACD
Ebenezer K. Aidoo et al [42]	2018	Kenya	Cross-sectional study	No	Yes	Yes	Yes	No	1-3-7
Michelle S. Hsiang et al [43]	2020	Namibia	Cluster randomised controlled trial	No	No	Yes	No	Yes	RACD

Munyaradzi Tambo et al [44]	2018	Namibia	Case-control study	No	No	Yes	No	No	RACD
Jennifer L Smith et al [45]	2017	Namibia	Case-control study	No	No	Yes	No	No	RACD
Megan Littrell et al [46]	2013	Senegal	Cross-sectional study	Yes	Yes	Yes	No	No	1-3-7
Ruben O. Conner et al [47]	2020	Senegal	Quasi-experimental study with control	No	Yes	Yes	Yes	Yes	1-3-7
Craig Davies et al [48]	2019	South Africa	Cohort study	Yes	No	No	No	No	1-3-7
Logan Stuck et al [49]	2020	Tanzania	Cross-sectional study	No	No	Yes	No	No	RACD
Tina van der Horst et al [50]	2020	Tanzania	Cross-sectional study	Yes	Yes	Yes	No	No	1-3-7
Benjamin Grossenbacher et al [51]	2020	Tanzania	Cross-sectional study	No	No	Yes	No	No	RACD
Eeshan Khandekar et al [52]	2019	Tanzania	Cross-sectional and qualitative study	No	Yes	Yes	No	No	1-3-7
Mkali H. R. et al [53]	2023	Tanzania	Cross-sectional study	Yes	Yes	Yes	Yes	No	RACD
Lynne Lohfeld et al [54]	2016	Zambia	Qualitative study	No	No	Yes	No	No	RACD
Kelly M. Searle et al [55]	2013	Zambia	Cross-sectional study	No	No	Yes	No	No	RACD
Joshua Yukich et al [56]	2017	Zambia	Cluster randomized controlled trial	No	No	Yes	No	Yes	RACD
Julia C Pringle et al [57]	2019	Zambia	Quasi-experimental study without control	No	No	Yes	No	No	RACD
Fiona R. P. Bhondoekhan et al [58]	2020	Zambia	Cross-sectional study	No	No	Yes	No	No	RACD
David A. Larsen et al [59]	2017	Zambia	Case-control study	No	Yes	Yes	No	No	1-3-7
David A. Larsen et al [60]	2015	Zambia	Quasi-experimental study without control	Yes	Yes	Yes	No	No	1-3-7
David A. Larsen et al [61]	2017	Zambia	Cohort study	No	No	Yes	No	No	RACD
Jessie Pinchoff et al [62]	2015	Zambia	Cross-sectional study	No	No	Yes	No	No	RACD
Daniel J. Bridges et al [63]	2020	Zambia	Cross-sectional study	No	Yes	Yes	Yes	No	1-3-7
Nakul Chitnis et al [64]	2019	Zambia	Cross-sectional study	No	No	Yes	No	No	RACD
Gillian H. Stresman et al [65]	2010	Zambia	Case-control study	No	No	Yes	No	No	RACD
Kelly M. Searle et al [66]	2016	Zambia	Cross-sectional study	No	No	Yes	No	No	RACD
Martin A. C et al [67]	2025	Zambia	Cluster randomized controlled trial	Yes	Yes	Yes	Yes	No	1-3-7
**South American Region**									
Pablo S. Fontoura et al [68]	2016	Brazil	Randomised controlled trial	No	No	Yes	No	No	RACD
Karen Molina Gomez et al [69]	2017	Colombia	Cross-sectional study	Yes	Yes	Yes	Yes	No	1-3-7

* Year = Year of publication

CN = Case notificaiton, CI = Case investigation, RACD = Ractive case detection, FI = Focus investigation, FR = Focus response, 1-3-7 = 1-3-7 reactive surveillance and response strategy, 2-7 = 2-7 reactive surveillance and response strategy

Yes = The paper reports respective data of CN, CI, RACD, FI or FR, No = The paper doesn’t report respective data of CN, CI, RACD, FI or FR

Eswatini was formerly known as Swaziland until 2018

Supplementary Table 2: Implementation of reactive case detection

Author and year of publication	Country of research	Implementation period (in months)	Malaria diagnostic test used	Time schedule* (in days)	Radius in meter and/or other criteria of screening
			**Asia-Pacific Region**		
Feng 2016	China	120	RDT + Microscopy + PCR	7	.
Feng 2018	China	72	.	7	.
Wang 2017	China	36	RDT + Microscopy	7	Whole village
Xiao 2015	China	.	PCR and Microscopy	7	300
Zhou 2015	China	18	PCR	7	.
Zhang 2024	China	.	.	7	.
Cotter 2017	China, Indonesia, Thailand	3	RDT + Microscopy + PCR	7	100
Hustedt 2016	Cambodia	11	RDT and PCR	3	10 nearest neighbouring households
Kheang 2020	Cambodia	18	RDT	7	Nearest neighbouring households and co-travellers
Lek 2020	Cambodia	24	RDT and PCR	14	.
Rossi 2018	Cambodia	18	RDT and PCR	.	Index households and co-exposed persons
Htike 2024	Lao PDR	.	RDT + Microscopy + PCR	7	Index households and neighbouring households
Rogawski 2012	Thailand	1	Microscopy and PCR	14	residents in 1000 and MMPs
Ngo 2019	Vietnam	24	RDT	3	Whole village
Oo 2023	Vietnam	.	RDT + Microscopy + PCR	7	Index households and neighbouring households
Wangdi 2016	Bhutan	8	RDT + Microscopy	.	1000
Herdiana 2016	Indonesia	18	Microscopy and PCR	7	500
Zelman 2018	Indonesia	20	Microscopy and PCR and LAMP	7	7 nearest neighbouring households (300 – 500)
Baliga 2019	India	60	RDT + Microscopy	.	Whole city
Donald 2016	Vanuatu	1	RDT and PCR	5	500
			**African Region**		
Bekolo 2019	Cameroon	3	RDT	.	Index household
Hamze 2016	Democratic Republic of Congo	2	RDT	.	.
Bansil 2018	Ethiopia	5	RDT	.	100
Tessema 2020	Ethiopia	3	RDT and PCR	2	6 nearest neighboring households
Zemene 2018	Ethiopia	6	Microscopy and PCR	7	200
Hsiang 2020	Eswatini	31	RDT and LAMP	35	500
Sturrock 2013	Eswatini	31	RDT	.	1000
Aidoo 2018	Kenya	12	Microscopy and PCR	7	100

Hsiang 2020	Namibia	12	RDT	35	500
Smith 2017	Namibia	18	RDT and LAMP	2	4 nearest neighbour households
Tambo 2018	Namibia	20	RDT and PCR and LAMP	.	4 nearest neighbour households
Conner 2020	Senegal	4	RDT	.	100
Littrell 2013	Senegal	3	RDT	.	5 nearest neighbouring households (300 – 500 M)
Grossenbacher 2020	Tanzania	14	RDT and PCR	.	200
Stuck 2020	Tanzania	12	RDT and PCR	.	9 nearest neighbouring households
VanderHorst 2020	Tanzania	24	RDT	3	Index households
Mkali 2023	Tanzania	120	RDT	7	Index households
Bhondoekhan 2020	Zambia	31	RDT or PCR	.	250
Bridges 2020	Zambia	60	RDT + PCR	7	9 nearest neighbouring households
Larsen 2015	Zambia	12	RDT	7	140
Larsen 2017	Zambia	24	RDT	.	140
Pinchoff 2015	Zambia	13	RDT	.	Index household
Pringle 2019	Zambia	16	RDT	7	250
Searle 2016	Zambia	12	RDT	7	140
Stresman 2010	Zambia	3	RDT and PCR	14	Index households
Martin 2025	Zambia	.	RDT	7	140
**South American Region**					
Molina Gómez 2017	Colombia	1	PCR	.	4 nearest neighbour households
Molina Gómez 2017	Colombia	1	Microscopy	.	4 nearest neighbour households
Fontoura 2016	Brazil	7	Microscopy	.	3000
Fontoura 2016	Brazil	7	PCR	.	3000

* Time schedule to complete RACD in days
. Missing data
LAMP – Loop-Mediated Isothermal Amplification, PCR – Polymerase Chain Reaction, RDT – Rapid Diagnostic Test
MMP – Mobile and migrant populations
Eswatini was formerly known as Swaziland until 2018

Study-specific and geographical region pooled estimates

Case notification

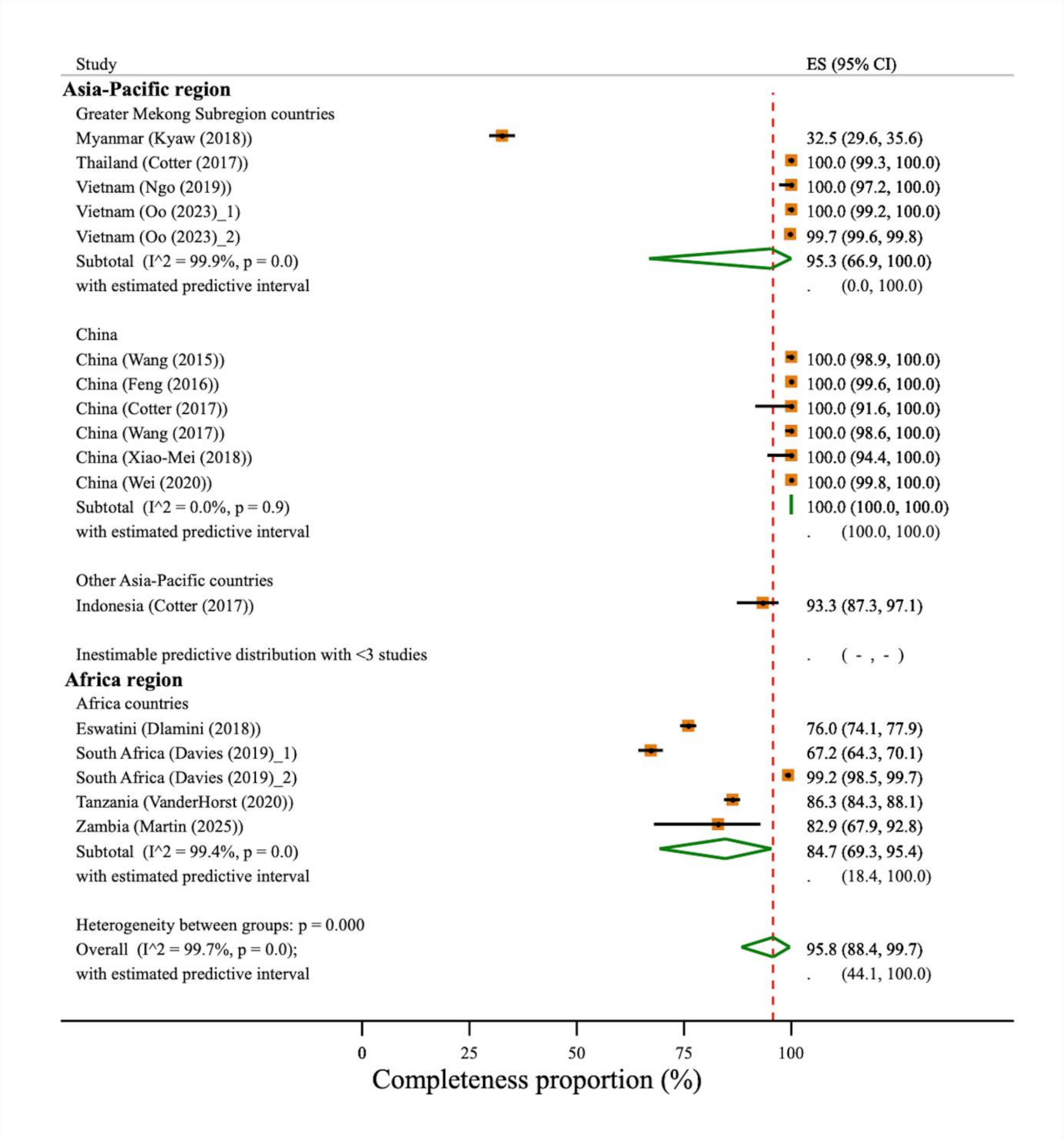

Study estimates and pooled proportion using DerSimonian and Laird random effect with Freeman-Tukey Double Arcsine Transformation
Red-dotted line – overall completeness of case notification
ES – Estimated proportion
Predictive interval is inestimable for Greater Mekong Subregion due to very large between-study variance (τ^2)
Davies (2019)_1, Oo (2023)_1 - Online or web-based notification
Davies (2019)_2, Oo (2023)_2 - Paper-based notification

Supplementary Fig 1. Completeness of malaria case notification stratified by geographical region

Case investigation and classification

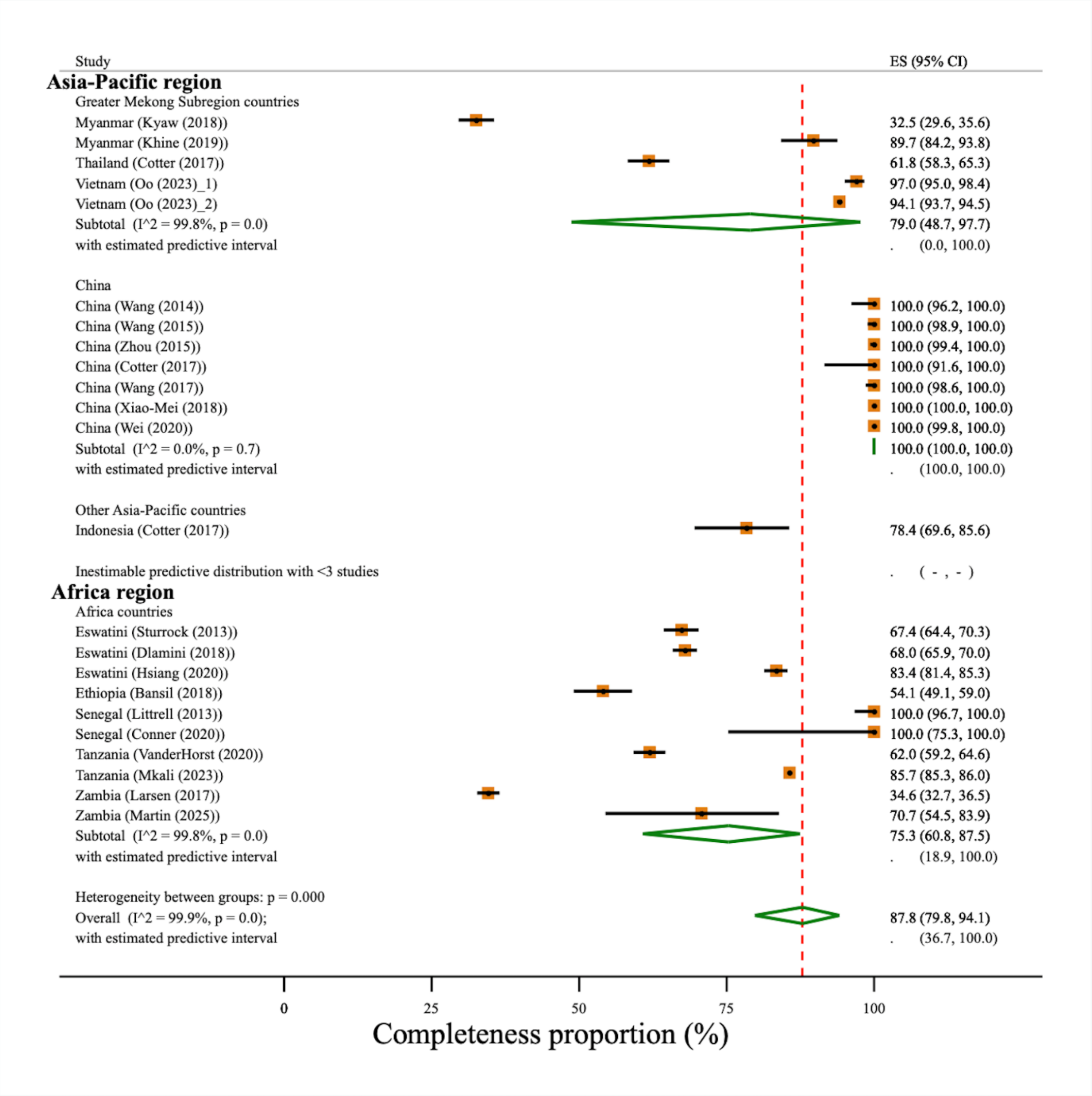

Study estimates and pooled proportion using DerSimonian and Laird random effect with Freeman-Tukey Double Arcsine Transformation
Red-dotted line – overall completeness of case investigation and classification
ES – Estimated proportion
Predictive interval is inestimable for Greater Mekong Subregion due to very large between-study variance (τ^2)
Oo (2023)_1 – CI must be completed within 2 days
Oo (2023)_2 – CI must be completed within 3 days

Supplementary Figure 2. Completeness of case investigation and classification stratified by geographical region

Reactive case detection

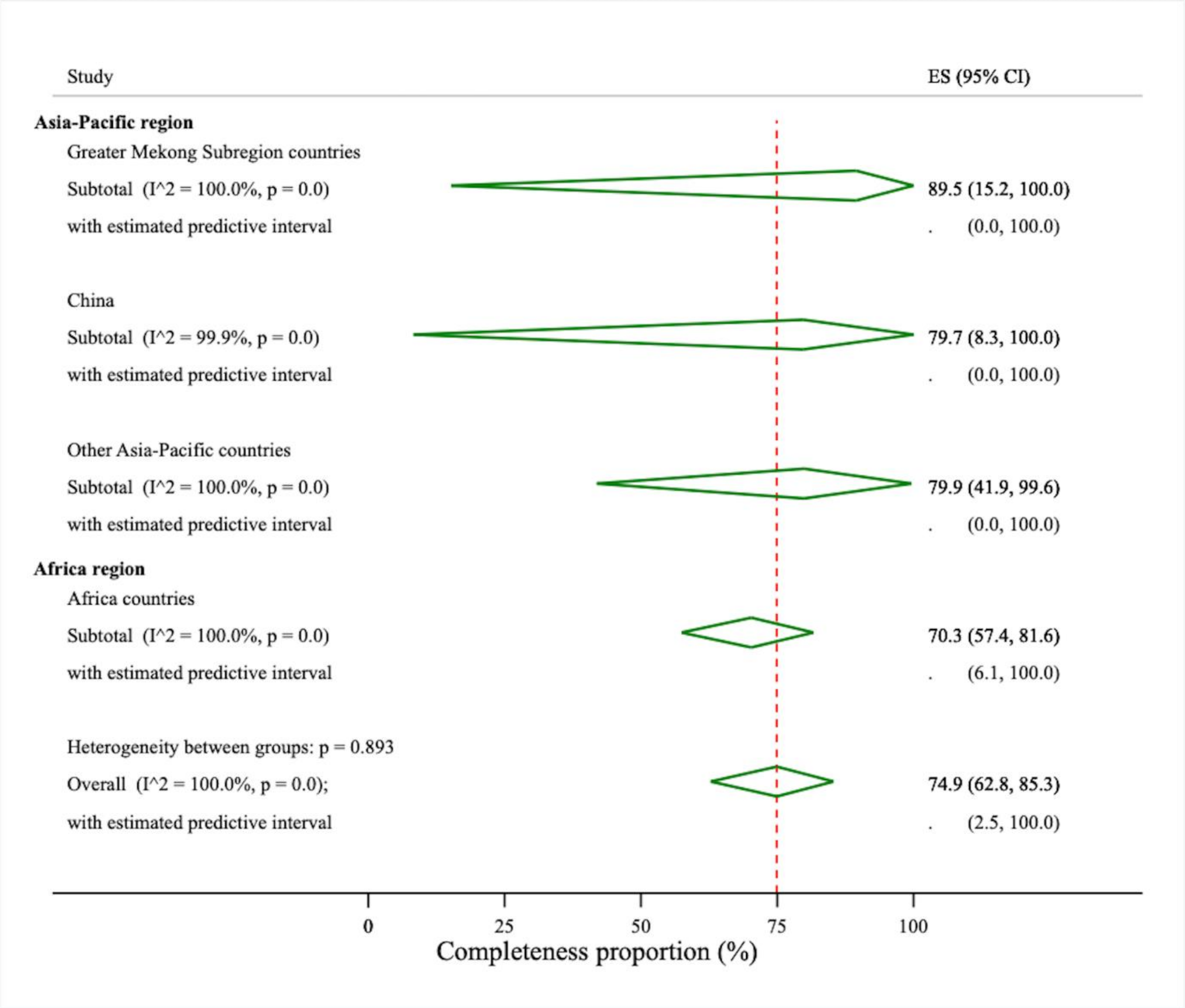

Study estimates and pooled proportion using DerSimonian and Laird random effect with Freeman-Tukey Double Arcsine Transformation
Red-dotted line – overall completeness of Reactive Case Detection (RACD)
ES – Estimated proportion

Supplementary Figure 3. Completeness of RACD stratified by geographical region

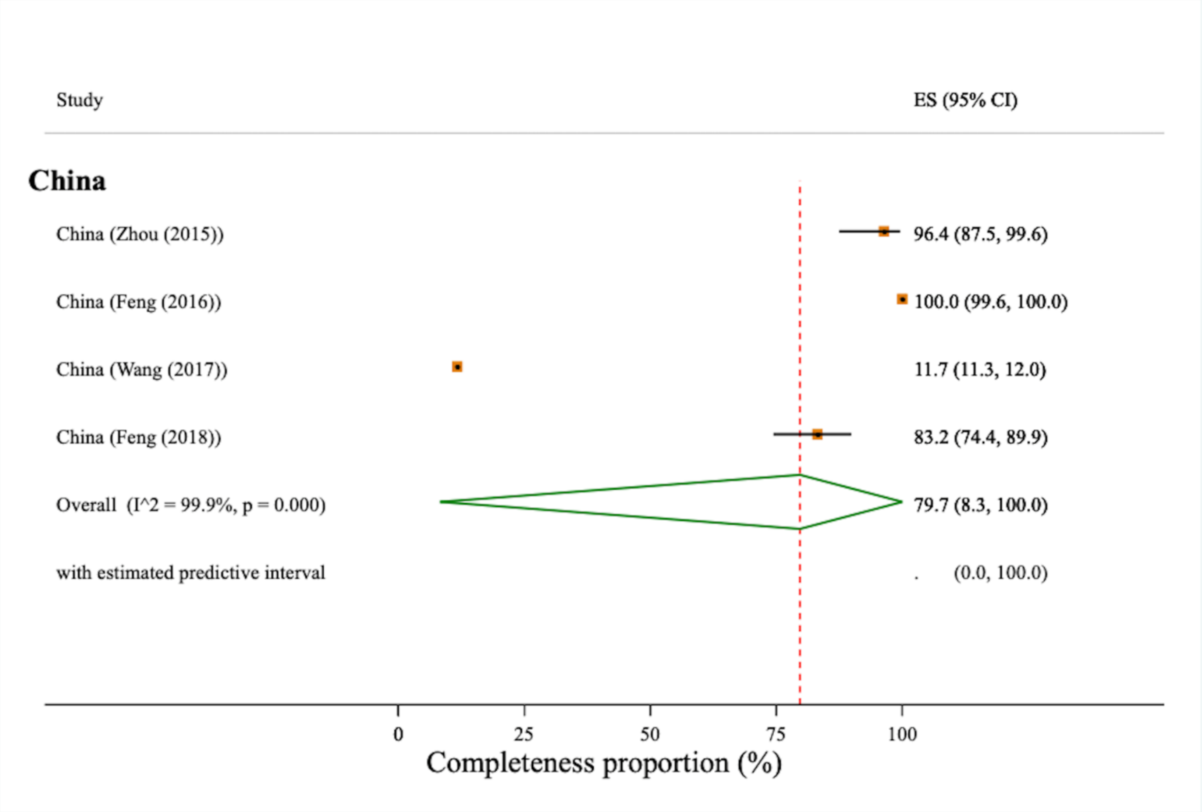

Study estimates and pooled proportion using DerSimonian and Laird random effect with Freeman-Tukey Double Arcsine Transformation
Red-dotted line – overall completeness of RACD
ES – Estimated proportion

Supplementary Figure 3a. Completeness of RACD in China

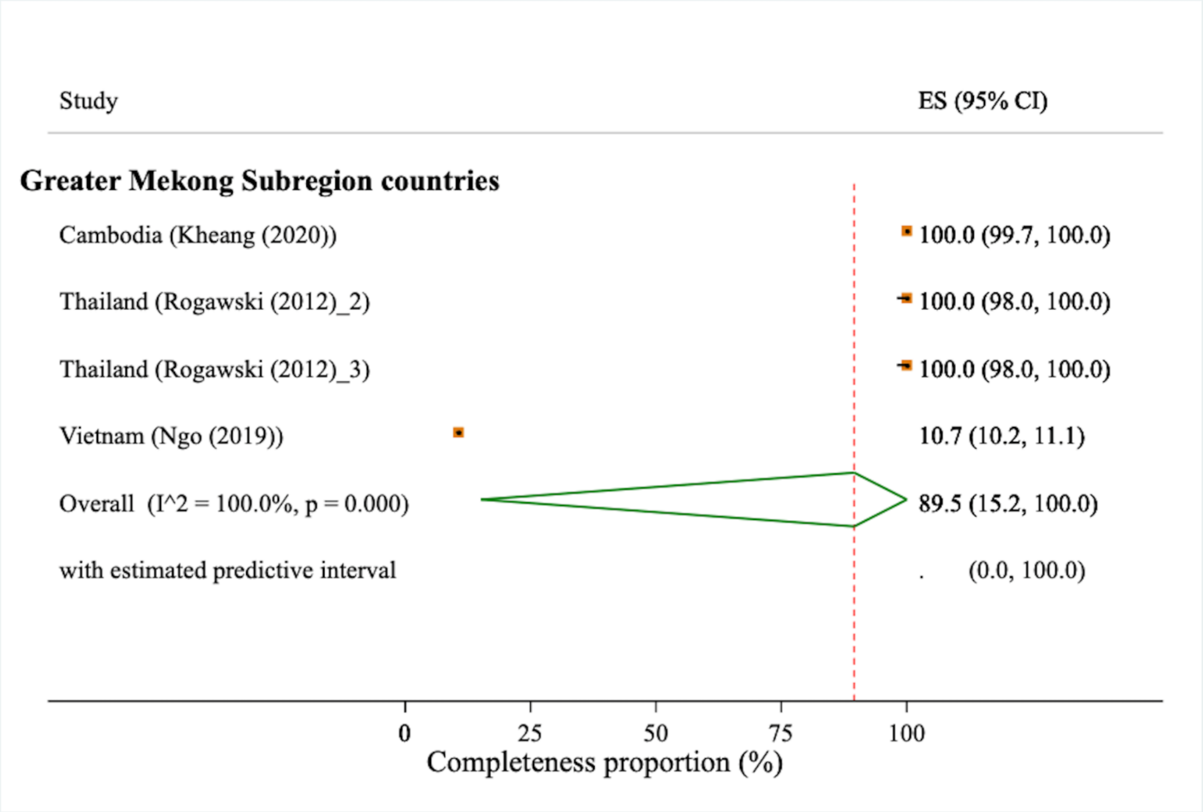

Study estimates and pooled proportion using DerSimonian and Laird random effect with Freeman-Tukey Double Arcsine Transformation
Red-dotted line – overall completeness of RACD
ES – Estimated proportion
Rogawski (2012)_2 – used microscopy for RACD
Rogawski (2012)_3 – used PCR for RACD

Supplementary Figure 3b. Completeness of RACD in Greater Mekong Subregion

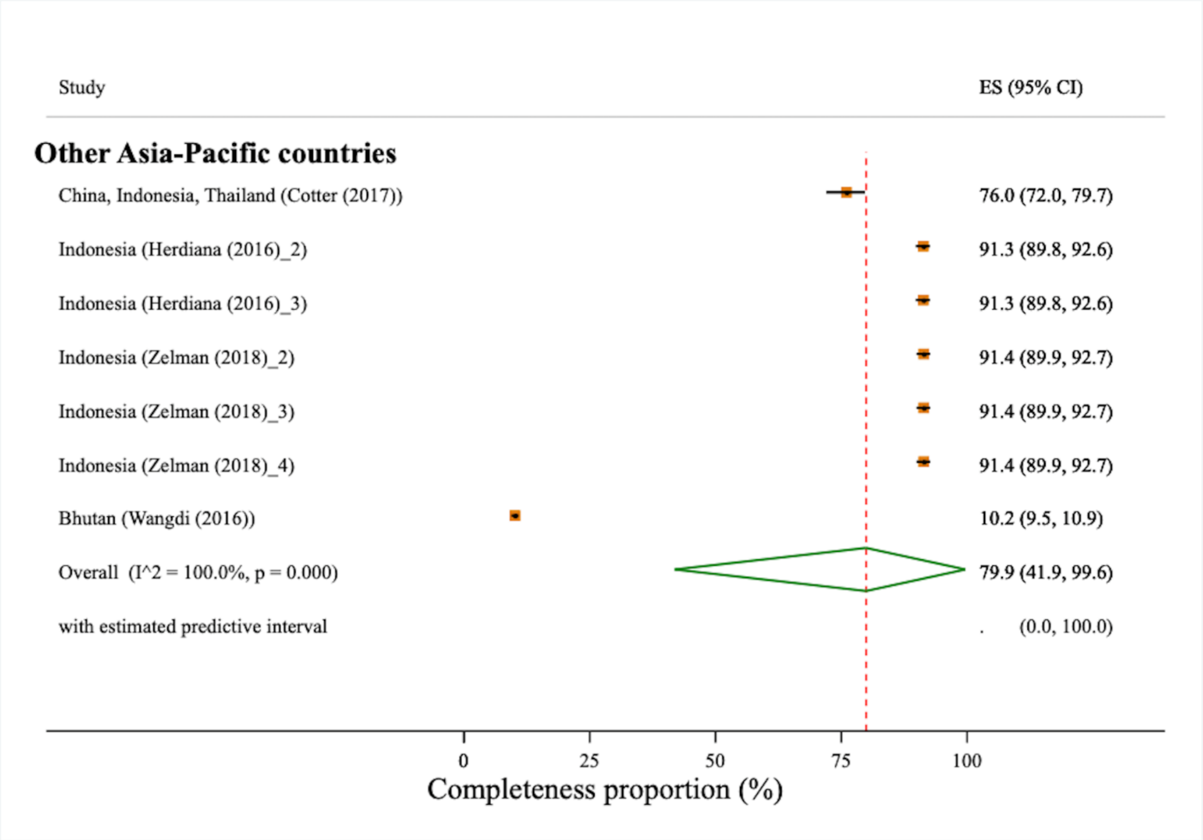

Study estimates and pooled proportion using DerSimonian and Laird random effect with Freeman-Tukey Double Arcsine Transformation
Red-dotted line – overall completeness of RACD
ES – Estimated proportion
Herdiana (2016)_2 and Zelman (2018)_2 – used microscopy for RACD
Herdiana (2016)_3 and Zelman (2018)_3 – used PCR for RACD
Zelman (2018)_4 – used LAMP for RACD

Supplementary Figure 3c. Completeness of RACD in other Asia-Pacific countries

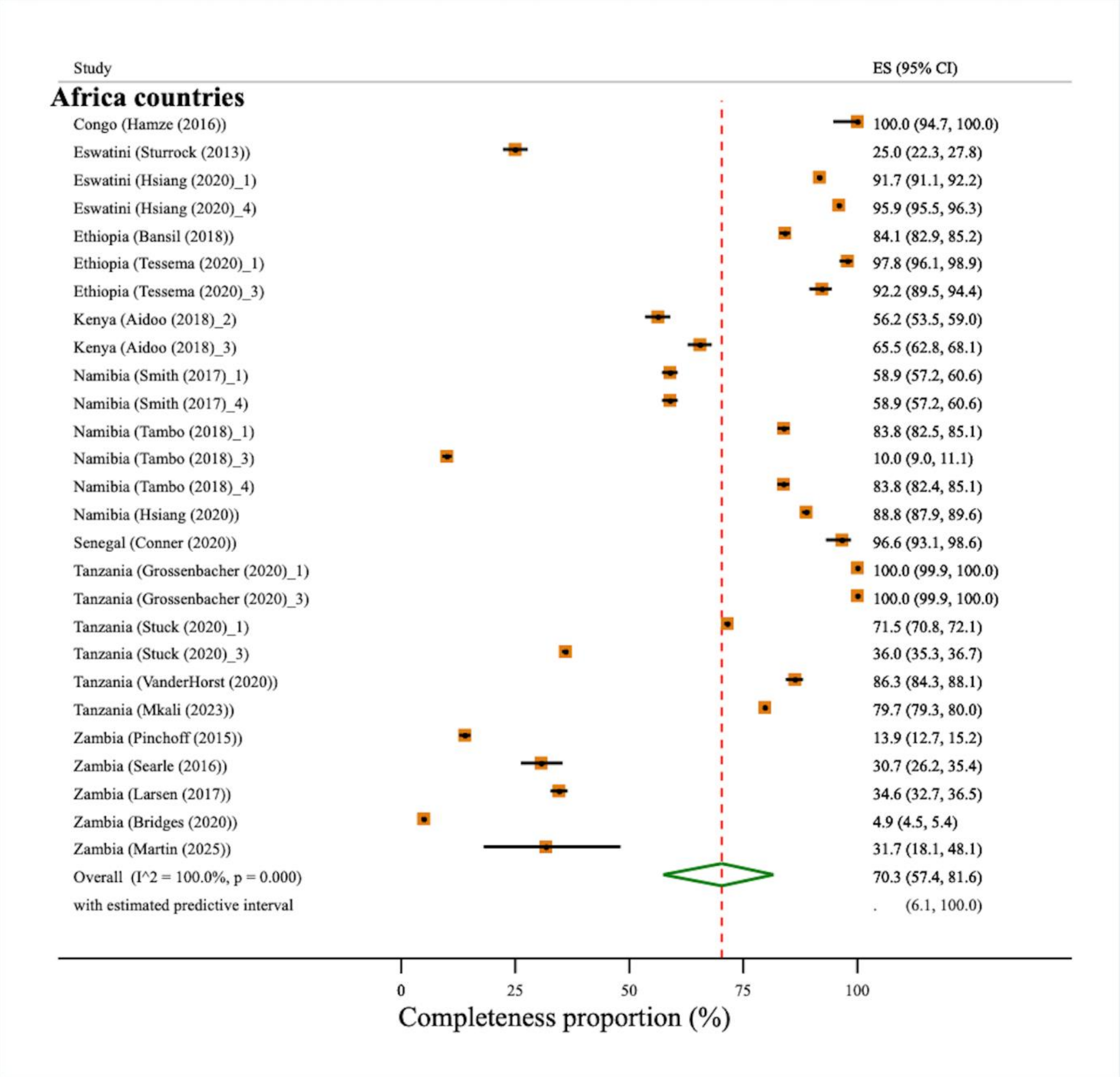

Study estimates and pooled proportion using DerSimonian and Laird random effect with Freeman-Tukey Double Arcsine Transformation

Red-dotted line – overall completeness of RACD

ES – Estimated proportion

Hsiang (2020)_1, Tessema (2020)_1, Smith (2017)_1, Tambo (2018)_1, Grossenbacher (2020)_1 and Stuck (2020)_1 – used RDT for RACD

Aidoo (2018)_2 – used microscopy for RACD

Tessema (2020)_3, Aidoo (2018)_3, Tambo (2018)_3, Grossenbacher (2020)_3 and Stuck (2020)_3 – used PCR for RACD

Hsiang (2020)_4, Smith (2017)_4 and Tambo (2018)_4 – used LAMP for RACD

Supplementary Figure 3d. Completeness of RACD in Africa region

References

1. Smith Gueye C, Sanders KC, Galappaththy GN, et al. Active case detection for malaria elimination: a survey among Asia Pacific countries. Malar J. 2013;12:358.
2. Wangdi K, Banwell C, Gatton ML, Kelly GC, Namgay R, Clements AC. Development and evaluation of a spatial decision support system for malaria elimination in Bhutan. Malar J. 2016;15:180.
3. Rossi G, Van den Bergh R, Nguon C, et al. Adapting Reactive Case Detection Strategies for falciparum Malaria in a Low-Transmission Area in Cambodia. Clin Infect Dis. 2018;66(2):296-8.
4. Kheang ST, Sovannaroth S, Barat LM, et al. Malaria elimination using the 1-3-7 approach: lessons from Sampov Loun, Cambodia. BMC Public Health. 2020;20(1):544.
5. Hustedt J, Canavati SE, Rang C, et al. Reactive case-detection of malaria in Pailin Province, Western Cambodia: lessons from a year-long evaluation in a pre-elimination setting. Malar J. 2016;15:132.
6. Lek D, Callery JJ, Nguon C, et al. Tools to accelerate falciparum malaria elimination in Cambodia: a meeting report. Malar J. 2020;19(1):151.
7. Wang D, Cotter C, Sun X, Bennett A, Gosling RD, Xiao N. Adapting the local response for malaria elimination through evaluation of the 1-3-7 system performance in the China-Myanmar border region. Malar J. 2017;16(1):54.
8. Lu G, Liu Y, Beiersmann C, Feng Y, Cao J, Müller O. Challenges in and lessons learned during the implementation of the 1-3-7 malaria surveillance and response strategy in China: a qualitative study. Infect Dis Poverty. 2016;5(1):94.
9. Zhou SS, Zhang SS, Zhang L, et al. China's 1-3-7 surveillance and response strategy for malaria elimination: Is case reporting, investigation and foci response happening according to plan? Infect Dis Poverty. 2015;4:55.
10. Wei C, Lu N, Yang R, Tang YR, Lü Q, Jiang JY. [Epidemic situation of malaria in Yunnan Province from 2014 to 2019]. Zhongguo Xue Xi Chong Bing Fang Zhi Za Zhi. 2020;32(5):483-8.
11. Feng J, Tu H, Zhang L, et al. Mapping transmission foci to eliminate malaria in the People's Republic of China, 2010-2015: a retrospective analysis. BMC Infect Dis. 2018;18(1):115.
12. Xiao HH, Liu J, Feng J, et al. [Screening Radius of Active Case Detection and the Malaria Parasite Rate of Carriers in China-Myanmar Border]. Zhongguo Ji Sheng Chong Xue Yu Ji Sheng Chong Bing Za Zhi. 2015;33(2):86-90.
13. Feng J, Liu J, Feng X, Zhang L, Xiao H, Xia Z. Towards Malaria Elimination: Monitoring and Evaluation of the "1-3-7" Approach at the China-Myanmar Border. Am J Trop Med Hyg. 2016;95(4):806-10.
14. Wang WM, Zhou HY, Liu YB, Cao YY, Cao J, Gao Q. [Establishment of malaria early warning system in Jiangsu Province III effect of automatic early warning information system on the response of malaria elimination]. Zhongguo Xue Xi Chong Bing Fang Zhi Za Zhi. 2014;26(1):27-31.
15. Wang WM, Zhou HY, Liu YB, Cao YY, Cao J, Gao Q. [Establishment of malaria early warning system in Jiangsu Province IV Implementation of key measures to eliminate malaria in Jiangsu Province in 2013]. Zhongguo Xue Xi Chong Bing Fang Zhi Za Zhi. 2015;27(2):134-8, 61.

16. Xiao-Mei H, Lai-Fu L, Wei-Ming W, Shi-Ying Z, Li-Zhong H, Liang J. [Evaluation of malaria elimination surveillance in Liyang City from 2010 to 2016]. Zhongguo Xue Xi Chong Bing Fang Zhi Za Zhi. 2018;30(5):559-62.
17. Zhang L, Yin J, Xia Z. Analysis of effectiveness and challenges in preventing the re-establishment of malaria transmission in China. China Tropical Medicine. 2024;24(4):365-71.
18. Cotter C, Sudathip P, Herdiana H, et al. Piloting a programme tool to evaluate malaria case investigation and reactive case detection activities: results from 3 settings in the Asia Pacific. Malar J. 2017;16(1):347.
19. Baliga BS, Jain A, Koduvattat N, et al. Indigenously developed digital handheld Android-based Geographic Information System (GIS)-tagged tablets (TABs) in malaria elimination programme in Mangaluru city, Karnataka, India. Malar J. 2019;18(1):444.
20. Zelman BW, Baral R, Zarlinda I, et al. Costs and cost-effectiveness of malaria reactive case detection using loop-mediated isothermal amplification compared to microscopy in the low transmission setting of Aceh Province, Indonesia. Malar J. 2018;17(1):220.
21. Herdiana H, Cotter C, Coutrier FN, et al. Malaria risk factor assessment using active and passive surveillance data from Aceh Besar, Indonesia, a low endemic, malaria elimination setting with Plasmodium knowlesi, Plasmodium vivax, and Plasmodium falciparum. Malar J. 2016;15(1):468.
22. US President's Malaria Initiative (PMI) IMP. LAOS 1-3-7 Malaria Elimination Strategy Assessment Report (Draft). Lao PDR: US President's Malaria Initiative (PMI), Impact Malaria Project; 2022.
23. Htike W, Win Han O, Aye Tun N, et al. Comprehensive evaluation of malaria reactive surveillance and response strategies in Lao People's Democratic Republic: a mixed-methods study. BMJ Open. 2024;14(8):e083060.
24. Kyaw AMM, Kathirvel S, Das M, et al. "Alert-Audit-Act": assessment of surveillance and response strategy for malaria elimination in three low-endemic settings of Myanmar in 2016. Trop Med Health. 2018;46:11.
25. Aung PP, Thein ZW, Hein ZNM, et al. Challenges in early phase of implementing the 1-3-7 surveillance and response approach in malaria elimination setting: A field study from Myanmar. Infect Dis Poverty. 2020;9(1):18.
26. Parker DM, Landier J, von Seidlein L, et al. Limitations of malaria reactive case detection in an area of low and unstable transmission on the Myanmar-Thailand border. Malar J. 2016;15(1):571.
27. Khine SK, Kyaw NTT, Thekkur P, Lin Z, Thi A. Malaria hot spot along the foothills of Rakhine state, Myanmar: geospatial distribution of malaria cases in townships targeted for malaria elimination. Trop Med Health. 2019;47:60.
28. Kandel S, Dahal G, Marasini RP, et al. Malaria reporting timeliness analysis and factors associated with delayed notification, 2018-2022, Nepal. PLOS Glob Public Health. 2024;4(8):e0003589.
29. Rogawski ET, Congpuong K, Sudathip P, et al. Active case detection with pooled real-time PCR to eliminate malaria in Trat province, Thailand. Am J Trop Med Hyg. 2012;86(5):789-91.
30. Khamsiriwatchara A, Sudathip P, Sawang S, et al. Artemisinin resistance containment project in Thailand. (I): Implementation of electronic-based malaria information system for early case detection and individual case management in provinces along the Thai-Cambodian border. Malar J. 2012;11:247.

31. Donald W, Pasay C, Guintran JO, et al. The Utility of Malaria Rapid Diagnostic Tests as a Tool in Enhanced Surveillance for Malaria Elimination in Vanuatu. PLoS One. 2016;11(11):e0167136.
32. Ngo TD, Canavati SE, Dinh HS, et al. Addressing operational challenges of combatting malaria in a remote forest area of Vietnam using spatial decision support system approaches. Geospat Health. 2019;14(2).
33. Win Han O, Nguyen XT, Ngo TVA, et al. Performance and feasibility of reactive surveillance and response strategies for malaria elimination in Vietnam: a mixed-methods study. Malar J. 2023;22(1):229.
34. Bekolo CE, Williams TD. Adding proactive and reactive case detection into the integrated community case management system to optimise diagnosis and treatment of malaria in a high transmission setting of Cameroon: an observational quality improvement study. BMJ Open. 2019;9(6):e026678.
35. Hamze H, Charchuk R, Jean Paul MK, Claude KM, Léon M, Hawkes MT. Lack of household clustering of malaria in a complex humanitarian emergency: implications for active case detection. Pathog Glob Health. 2016;110(6):223-7.
36. Hsiang MS, Ntshalintshali N, Kang Dufour MS, et al. Active Case Finding for Malaria: A 3-Year National Evaluation of Optimal Approaches to Detect Infections and Hotspots Through Reactive Case Detection in the Low-transmission Setting of Eswatini. Clin Infect Dis. 2020;70(7):1316-25.
37. Dlamini N, Zulu Z, Kunene S, et al. From diagnosis to case investigation for malaria elimination in Swaziland: is reporting and response timely? Public Health Action. 2018;8(Suppl 1):S8-s12.
38. Sturrock HJ, Novotny JM, Kunene S, et al. Reactive case detection for malaria elimination: real-life experience from an ongoing program in Swaziland. PLoS One. 2013;8(5):e63830.
39. Zemene E, Koepfli C, Tiruneh A, et al. Detection of foci of residual malaria transmission through reactive case detection in Ethiopia. Malar J. 2018;17(1):390.
40. Bansil P, Yeshiwondim AK, Guinovart C, et al. Malaria case investigation with reactive focal testing and treatment: operational feasibility and lessons learned from low and moderate transmission areas in Amhara Region, Ethiopia. Malar J. 2018;17(1):449.
41. Tessema SK, Belachew M, Koepfli C, et al. Spatial and genetic clustering of Plasmodium falciparum and Plasmodium vivax infections in a low-transmission area of Ethiopia. Sci Rep. 2020;10(1):19975.
42. Aidoo EK, Afrane YA, Machani MG, et al. Reactive case detection of Plasmodium falciparum in western Kenya highlands: effective in identifying additional cases, yet limited effect on transmission. Malar J. 2018;17(1):111.
43. Hsiang MS, Ntuku H, Roberts KW, et al. Effectiveness of reactive focal mass drug administration and reactive focal vector control to reduce malaria transmission in the low malaria-endemic setting of Namibia: a cluster-randomised controlled, open-label, two-by-two factorial design trial. Lancet. 2020;395(10233):1361-73.
44. Tambo M, Auala JR, Sturrock HJ, et al. Evaluation of loop-mediated isothermal amplification as a surveillance tool for malaria in reactive case detection moving towards elimination. Malar J. 2018;17(1):255.
45. Smith JL, Auala J, Tambo M, et al. Spatial clustering of patent and sub-patent malaria infections in northern Namibia: Implications for surveillance and response strategies for elimination. PLoS One. 2017;12(8):e0180845.
46. Littrell M, Sow GD, Ngom A, et al. Case investigation and reactive case detection for malaria elimination in northern Senegal. Malar J. 2013;12:331.

47. Conner RO, Dieye Y, Hainsworth M, et al. Mass testing and treatment for malaria followed by weekly fever screening, testing and treatment in Northern Senegal: feasibility, cost and impact. Malar J. 2020;19(1):252.
48. Davies C, Graffy R, Shandukani M, et al. Effectiveness of 24-h mobile reporting tool during a malaria outbreak in Mpumalanga Province, South Africa. Malar J. 2019;18(1):45.
49. Stuck L, Fakih BS, Al-Mafazy AH, et al. Malaria infection prevalence and sensitivity of reactive case detection in Zanzibar. Int J Infect Dis. 2020;97:337-46.
50. van der Horst T, Al-Mafazy AW, Fakih BS, et al. Operational Coverage and Timeliness of Reactive Case Detection for Malaria Elimination in Zanzibar, Tanzania. Am J Trop Med Hyg. 2020;102(2):298-306.
51. Grossenbacher B, Holzschuh A, Hofmann NE, et al. Molecular methods for tracking residual Plasmodium falciparum transmission in a close-to-elimination setting in Zanzibar. Malar J. 2020;19(1):50.
52. Khandekar E, Kramer R, Ali AS, et al. Evaluating Response Time in Zanzibar's Malaria Elimination Case-Based Surveillance-Response System. Am J Trop Med Hyg. 2019;100(2):256-63.
53. Mkali HR, Lalji SM, Al-Mafazy AW, et al. How Real-Time Case-Based Malaria Surveillance Helps Zanzibar Get a Step Closer to Malaria Elimination: Description of Operational Platform and Resources. Global Health Science and Practice. 2023;11(5).
54. Lohfeld L, Kangombe-Ngwenya T, Winters AM, et al. A qualitative review of implementer perceptions of the national community-level malaria surveillance system in Southern Province, Zambia. Malar J. 2016;15(1):400.
55. Searle KM, Shields T, Hamapumbu H, et al. Efficiency of household reactive case detection for malaria in rural Southern Zambia: simulations based on cross-sectional surveys from two epidemiological settings. PLoS One. 2013;8(8):e70972.
56. Yukich J, Bennett A, Yukich R, et al. Estimation of malaria parasite reservoir coverage using reactive case detection and active community fever screening from census data with rapid diagnostic tests in southern Zambia: a re-sampling approach. Malar J. 2017;16(1):317.
57. Pringle JC, Tessema S, Wesolowski A, et al. Genetic Evidence of Focal Plasmodium falciparum Transmission in a Pre-elimination Setting in Southern Province, Zambia. J Infect Dis. 2019;219(8):1254-63.
58. Bhondoekhan FRP, Searle KM, Hamapumbu H, et al. Improving the efficiency of reactive case detection for malaria elimination in southern Zambia: a cross-sectional study. Malar J. 2020;19(1):175.
59. Larsen DA, Ngwenya-Kangombe T, Cheelo S, et al. Location, location, location: environmental factors better predict malaria-positive individuals during reactive case detection than index case demographics in Southern Province, Zambia. Malar J. 2017;16(1):18.
60. Larsen DA, Chisha Z, Winters B, et al. Malaria surveillance in low-transmission areas of Zambia using reactive case detection. Malar J. 2015;14:465.
61. Larsen DA, Winters A, Cheelo S, et al. Shifting the burden or expanding access to care? Assessing malaria trends following scale-up of community health worker malaria case management and reactive case detection. Malar J. 2017;16(1):441.
62. Pinchoff J, Henostroza G, Carter BS, et al. Spatial patterns of incident malaria cases and their household contacts in a single clinic catchment area of Chongwe District, Zambia. Malar J. 2015;14:305.
63. Bridges DJ, Chishimba S, Mwenda M, et al. The use of spatial and genetic tools to assess Plasmodium falciparum transmission in Lusaka, Zambia between 2011 and 2015. Malar J. 2020;19(1):20.

64. Chitnis N, Pemberton-Ross P, Yukich J, et al. Theory of reactive interventions in the elimination and control of malaria. Malar J. 2019;18(1):266.
65. Stresman GH, Kamanga A, Moono P, et al. A method of active case detection to target reservoirs of asymptomatic malaria and gametocyte carriers in a rural area in Southern Province, Zambia. Malar J. 2010;9:265.
66. Searle KM, Hamapumbu H, Lubinda J, et al. Evaluation of the operational challenges in implementing reactive screen-and-treat and implications of reactive case detection strategies for malaria elimination in a region of low transmission in southern Zambia. Malar J. 2016;15(1):412.
67. Martin AC, Matoba J, Sing'anga C, et al. Implementation outcomes of 1-3-7 focus investigation for malaria in a low transmission setting in Southern Province, Zambia: A mixed methods study. PLOS GLOBAL PUBLIC HEALTH. 2025;5(1).
68. Fontoura PS, Finco BF, Lima NF, et al. Reactive Case Detection for Plasmodium vivax Malaria Elimination in Rural Amazonia. PLoS Negl Trop Dis. 2016;10(12):e0005221.
69. Molina Gómez K, Caicedo MA, Gaitán A, et al. Characterizing the malaria rural-to-urban transmission interface: The importance of reactive case detection. PLoS Negl Trop Dis. 2017;11(7):e0005780.
