# Supplementary material for: Implementation and fidelity of reactive surveillance and response strategies for malaria elimination: a systematic review and meta-analysis
Source: BMJ Public Health. 2025 Nov 13;3(2):e001180. doi: 10.1136/bmjph-2024-001180 (PMC12625913; doi:10.1136/bmjph-2024-001180)
Supplement: online supplemental file 6 [file bmjph-3-2-s006.docx]

# Supplementary material 6: Detail search strategy

## Search strategy and syntax of online databases

1. PubMed

((malaria[Title/Abstract] OR plasmodium[Title/Abstract] OR vivax[Title/Abstract] OR falciparum[Title/Abstract]) AND ("reactive surveillance" OR "case detection" OR "foci investigation" OR "RACD" OR "1-3-7"))

<https://pubmed.ncbi.nlm.nih.gov/?term=%28%28malaria%5BTitle%2FAbstract%5D+OR+plasmodium%5BTitle%2FAbstract%5D+OR+vivax%5BTitle%2FAbstract%5D+OR+falciparum%5BTitle%2FAbstract%5D%29+AND+%28%22reactive+surveillance%22+OR+%22case+detection%22+OR+%22foci+investigation%22+OR+%22RACD%22+OR+%221-3-7%22%29%29&sort=>

1. Web of Science

(ALL=(malaria OR plasmodium OR vivax OR falciparum)) AND ALL=("reactive surveillance" OR "case detection" OR "foci investigation" OR "RACD" OR "1-3-7")

<https://www.webofscience.com/wos/woscc/summary/67b32017-41aa-48ce-a0c5-686d7d798e25-017ed05abb/relevance/1>

1. Scopus

( TITLE-ABS-KEY ( malaria OR plasmodium OR vivax OR falciparum ) AND TITLE-ABS-KEY ( "reactive surveillance" OR "case detection" OR "foci investigation" OR "RACD" OR "1-3-7" ) )

<https://www.scopus.com/results/results.uri?st1=malaria+OR+plasmodium+OR+vivax+OR+falciparum&st2=%22reactive+surveillance%22+OR+%22case+detection%22+OR+%22foci+investigation%22+OR+%22RACD%22+OR+%221-3-7%22&s=%28TITLE-ABS-KEY%28malaria+OR+plasmodium+OR+vivax+OR+falciparum%29+AND+TITLE-ABS-KEY%28%22reactive+surveillance%22+OR+%22case+detection%22+OR+%22foci+investigation%22+OR+%22RACD%22+OR+%221-3-7%22%29%29&limit=10&origin=resultslist&sort=plf-f&src=s&sot=b&sdt=cl&sessionSearchId=1bb21c70c4866446ddd5643a610b4ae6>

1. African Index Medicus

tw:((tw:(malaria OR plasmodium OR vivax OR falciparum)) AND (tw:(reactive surveillance OR case detection OR foci investigation OR "RACD" OR "1-3-7")))

<https://search.bvsalud.org/aimafro/?output=site&lang=en&from=1&sort=YEAR_ASC&format=summary&count=20&fb=&page=1&tab=&range_year_start=&range_year_end=&skfp=&index=tw&q=%28tw%3A%28malaria+OR+plasmodium+OR+vivax+OR+falciparum%29%29+AND+%28tw%3A%28reactive+surveillance+OR+case+detection+OR+foci+investigation+OR+%22RACD%22+OR+%221-3-7%22%29%29>

1. Latin America And The Caribbean Literature On Health Sciences (LILACS)

(malaria OR plasmodium OR vivax OR falciparum) AND (reactive surveillance OR case detection OR foci investigation OR "RACD" OR "1-3-7") AND db:("LILACS")

<https://pesquisa.bvsalud.org/portal/?output=site&lang=en&from=1&sort=YEAR_DESC&format=summary&count=20&fb=&page=1&tab=2&filter%5Bdb%5D%5B%5D=LILACS&range_year_start=&range_year_end=&skfp=&index=&q=%28malaria+OR+plasmodium+OR+vivax+OR+falciparum%29+AND+%28reactive+surveillance+OR+case+detection+OR+foci+investigation+OR+%22RACD%22+OR+%221-3-7%22%29>

## Sources of grey literature searches

- UN agencies (World Health Organization <http://www.who.int/en/> , UNICEF <http://www.unicef.org/> , UNOPS <https://www.unops.org/english/Pages/Home.aspx> );
- International Organizations (IO) and Non-Government Organizations (NGO) (Population Services International <http://www.psi.org/> , Malaria Consortium <http://www.malariaconsortium.org/> , International Organizations for Migration <http://www.iom.int/> , Save the Children International <https://www.savethechildren.net/> , Médecins Sans Frontières (MSF) International <http://www.msf.org/> , Community Partners International <http://cpintl.org/> );
- Philanthropies and donor agencies (Bill and Malinda Gate Foundation <http://www.gatesfoundation.org/> , United States Agency for International Development <https://www.usaid.gov/> , UK Department for International Development <https://www.gov.uk/government/organisations/department-for-international-development> , Australian Department of Foreign Affairs and Trade <http://dfat.gov.au/pages/default.aspx> , Asia Development Bank <http://www.adb.org/> , Japan International Cooperation Agency <http://www.jica.go.jp/english/> ).
- [OpenGrey ([www.opengrey.eu](http://www.opengrey.eu) ; to date of search)]
- [Agency for Healthcare Research and Quality (AHRQ; [www.ahrq.gov](http://www.ahrq.gov) ; to date of search)]
- [National Institute for Health and Clinical Excellence (NICE; [www.nice.org.uk](http://www.nice.org.uk) ; to date of search).]
